# Supplementary material for: Composition and metabolism of microbial communities in soil pores
Source: Nat Commun. 2024 Apr 27;15:3578. doi: 10.1038/s41467-024-47755-x (PMC11055953; doi:10.1038/s41467-024-47755-x)
Supplement: Supplementary file 1 — Supplementary Information [file 41467_2024_47755_MOESM1_ESM.pdf]

Title: **Composition and metabolism of microbial communities in soil pores**

Authors: **Zheng Li[1], Alexandra N. Kravchenko[2,3*], Alison Cupples[1], Andrey K. Guber[2,3], Yakov Kuzyakov[4], G. Philip Robertson[3,5], and Evgenia Blagodatskaya[6]**

[1] Department to Civil and Environmental Engineering, Michigan State University, East Lansing MI, USA 48824

[2] Department of Plant, Soil and Microbial Sciences, Michigan State University, East Lansing MI, USA 48824

[3] DOE Great Lakes Bioenergy Research Center, Michigan State University, East Lansing, MI, USA

[4] Department of Soil Science of Temperate Ecosystems, Department of Agricultural Soil Science, University of Göttingen, Göttingen, Germany

[5] W. K. Kellogg Biological Station, Michigan State University, Hickory Corners, MI 49060

[6] Helmholtz Centre for Environmental Research, Halle, Germany

*Corresponding author:

Email: kravche1@msu.edu

Phone: (01) 517.353.0469

This file includes:

Figures S1-S8

Tables S1-S15

Figure S1. a) A schematic representation of the sampling scheme and types of samples employed at different stages of the study. **b)** Experimental set up used in the glucose addition experiment. Shown is a set of five chambers each holding one of the 5 intact soil cores from five glucose application treatments representing two glucose sources, ^{12}C or ^{13}C, added to either small or large pores, and one control core with water added to pores of both sizes.

**a)**	**Bare soil**		**Switchgrass**		**Prairie**	
Field plots per vegetation system	4		3		3	
Intact 5x5 cm cores per field plot 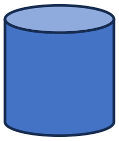	6		6		6	
Intact 5x5 cm cores used for X-ray µCT (2 micro-cores) 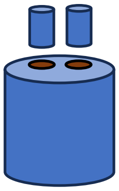	1		1		1	
Intact 5x5 cm cores used for ^{13}C glucose addition experiment 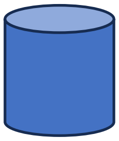	5: 1) ^{12}C in small pores 2) ^{12}C in large pores, 3) ^{13}C in small pores 4) ^{13}C in large pores, 5) water only control		5: 1) ^{12}C in small pores 2) ^{12}C in large pores, 3) ^{13}C in small pores 4) ^{13}C in large pores, 5) water only control		5: 1) ^{12}C in small pores 2) ^{12}C in large pores, 3) ^{13}C in small pores 4) ^{13}C in large pores, 5) water only control	
Intact 5x5 cm cores split in half for 24 hr and 30 d soil ^{13}C and SIP analyses 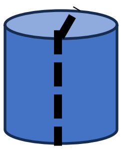	24 hr	30d	24 hr	30d	24 hr	30d
	5	Lost during COVID19 lab closure	5	5	5	5

b)

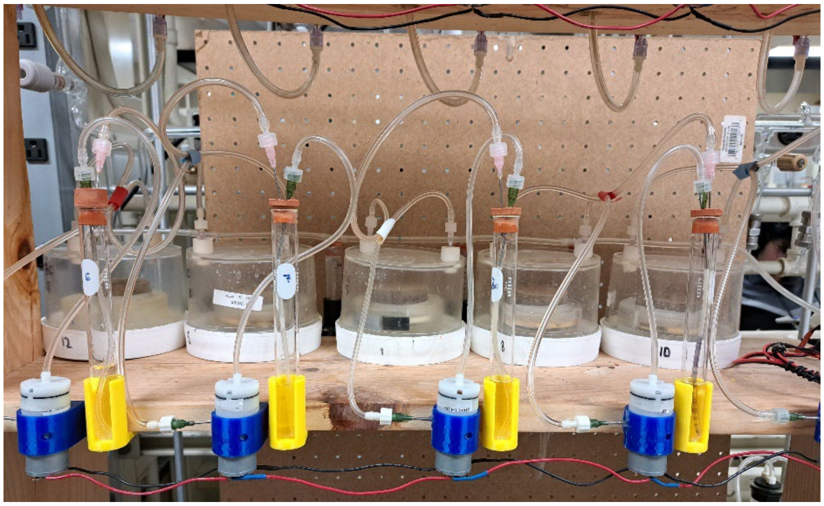

Figure S2. Saturation of the pores by the liquid added to small (orange) or large (blue) pores. Shown are means and standard deviations (n=3). Shaded areas mark the target small (orange) and large (blue) pores. The volumes of the small (4-10 µm Ø) and large (30-150 µm Ø) pores, i.e., the pore classes targeted for the glucose addition, constituted ~1.6% and ~4.4% of the total soil volume, respectively.

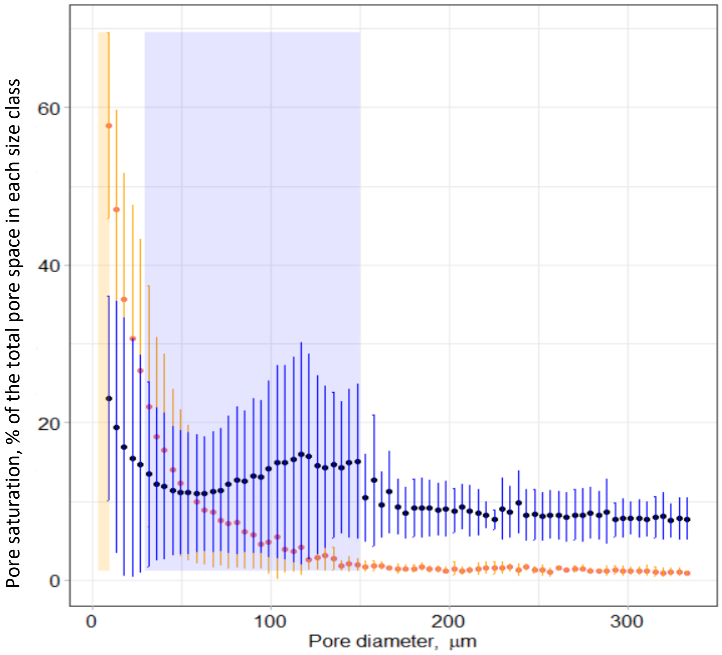

Figure S3. Principal Coordinate Analysis (PCoA) plots across two incubation times.

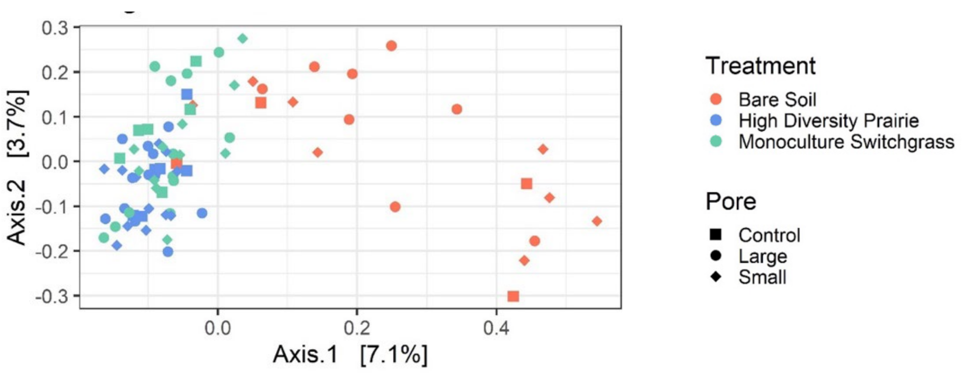

Figure S4. Relative abundances of the most abundant (a) phyla, (b) genera, (c) orders for the three studied plant systems, and d) genera that were found to be ^{13}C enriched during the studied incubation times

a)

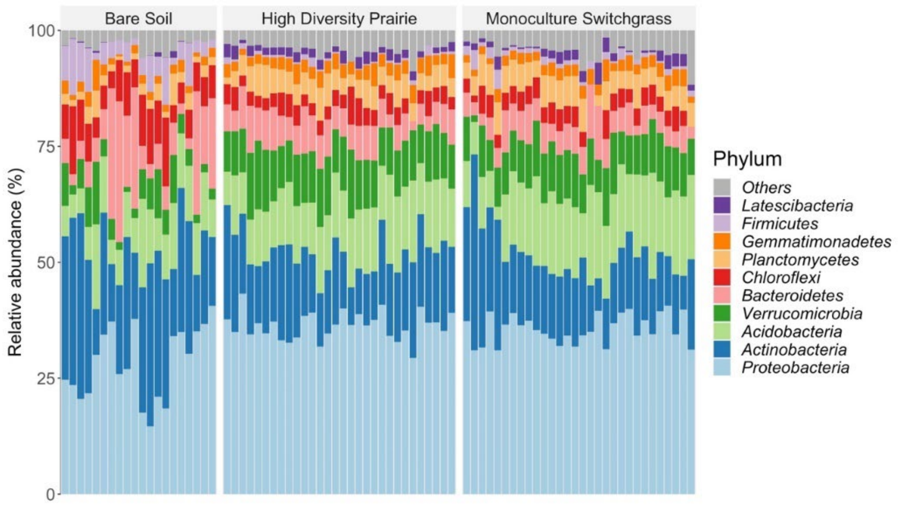

b)

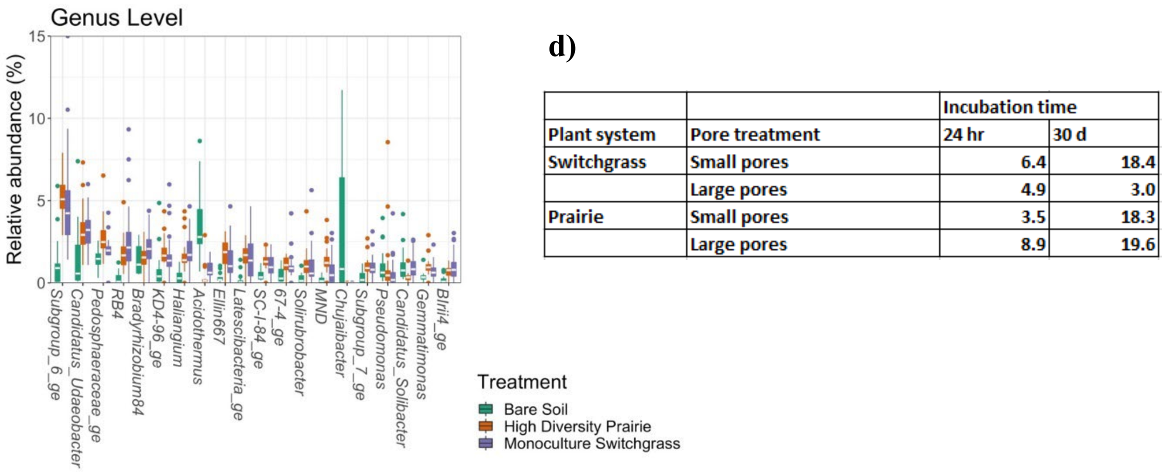

c)

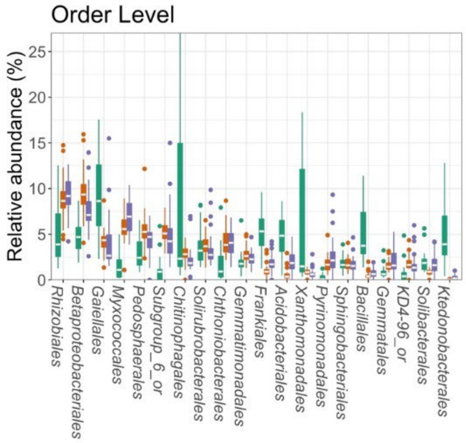

d)

		Incubation time	
**Plant system**	**Pore treatment**	**24 hr**	**30 d**
**Switchgrass**	**Small pores**	**6.4**	**18.4**
	**Large pores**	**4.9**	**3.0**
**Prairie**	**Small pores**	**3.5**	**18.3**
	**Large pores**	**8.9**	**19.6**

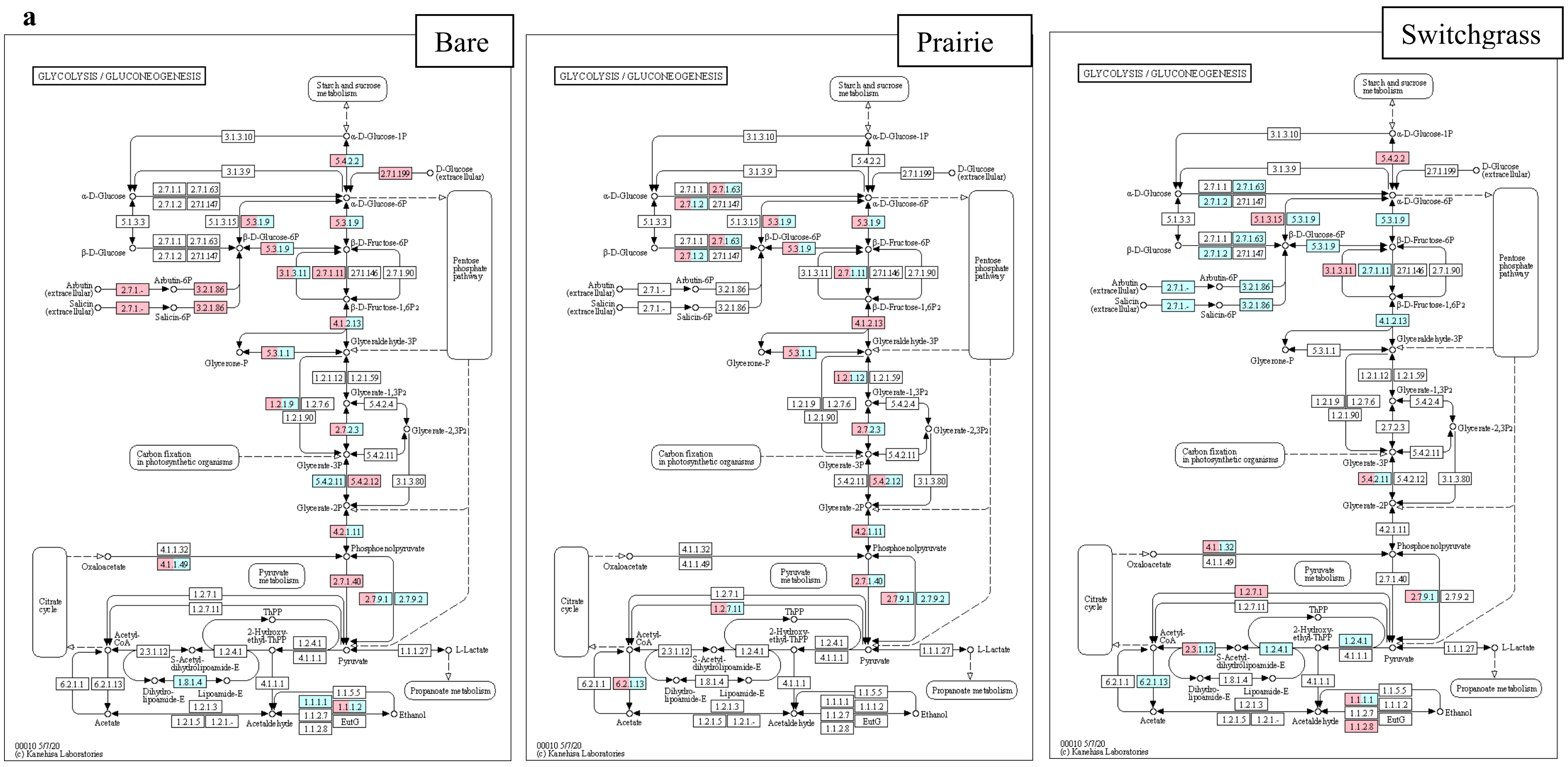
a
Bare
Prairie
Switchgrass
GLYCOLYSIS / GLUCONEOGENESIS

b

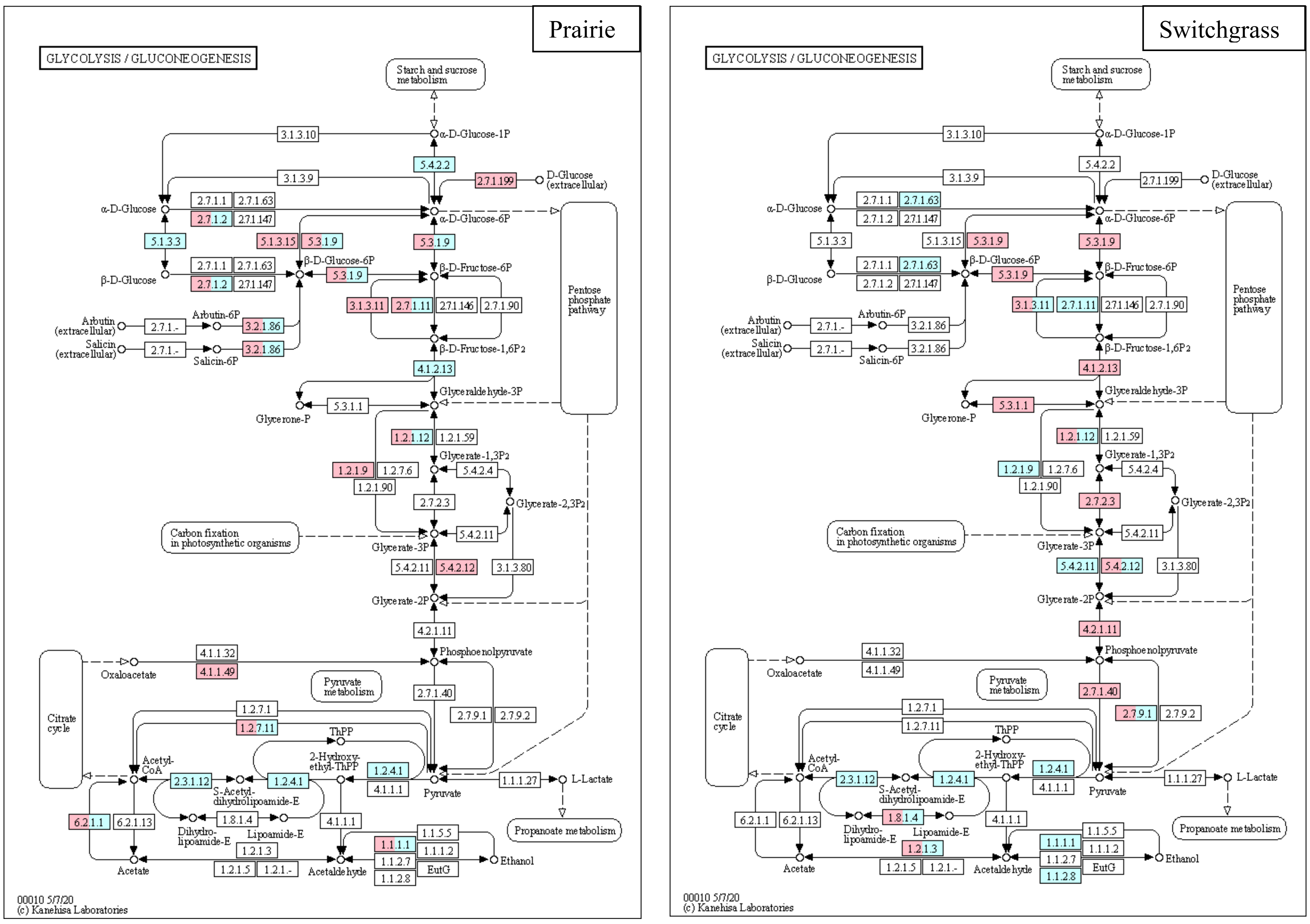

Figure S5. Genes enriched in the glycolysis/gluconeogenesis pathway in the studied plant systems at 24-hour (a) and 30-day incubations (b). Blue and pink mark enrichments in small and large pores, respectively. Diagram shown with KEGG Copyright Permission 230979.

a

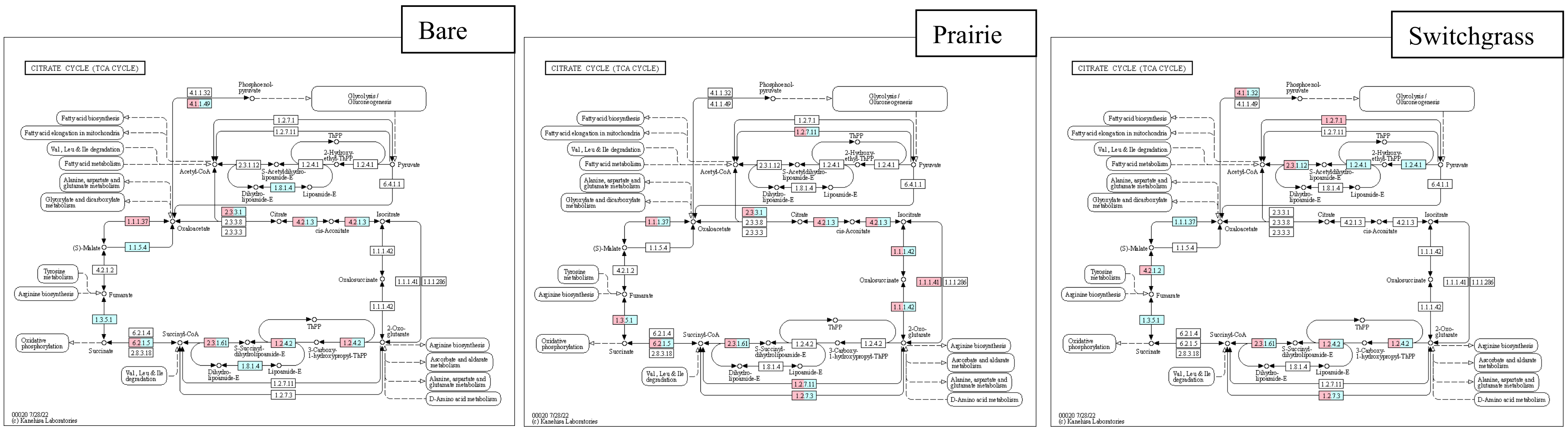

b

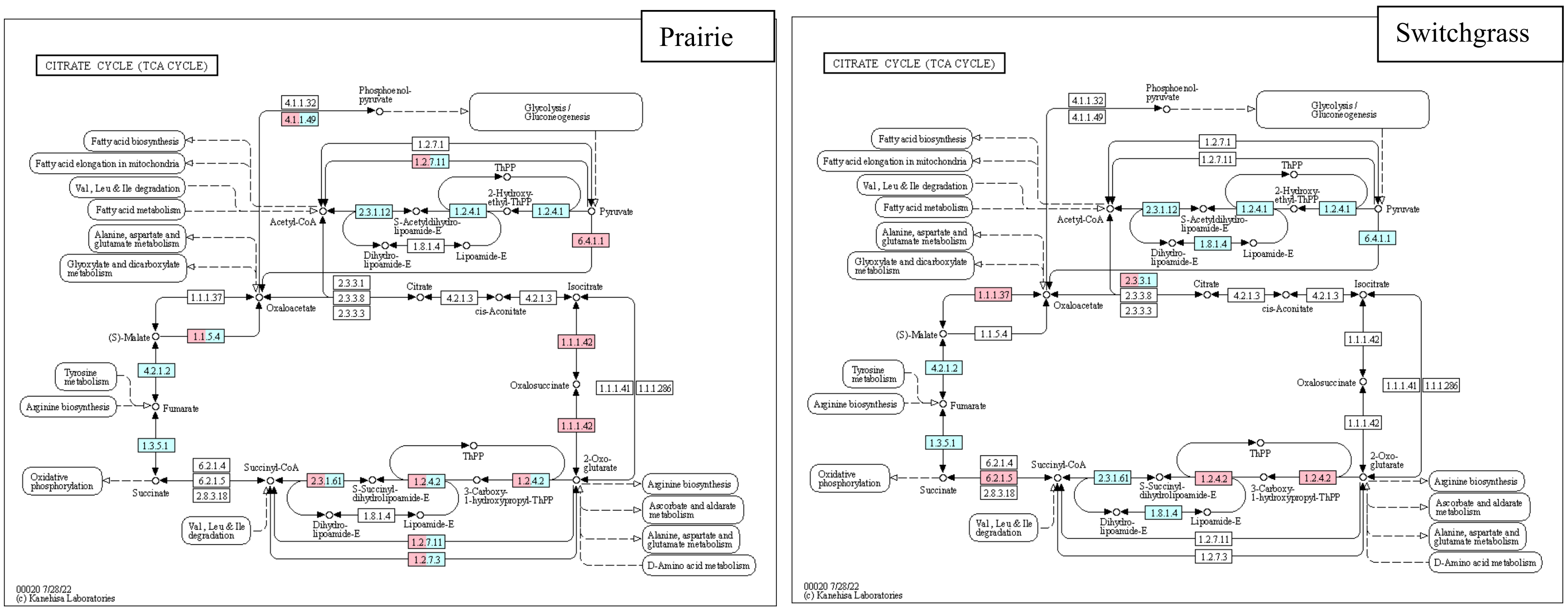

Figure S6. Genes enriched in the citrate (TCA) cycle in the studied plant systems at 24-hour (a) and 30-day incubations (b). Blue and pink mark enrichments in small and large pores, respectively. Diagrams shown with KEGG Copyright Permission 230979.

a

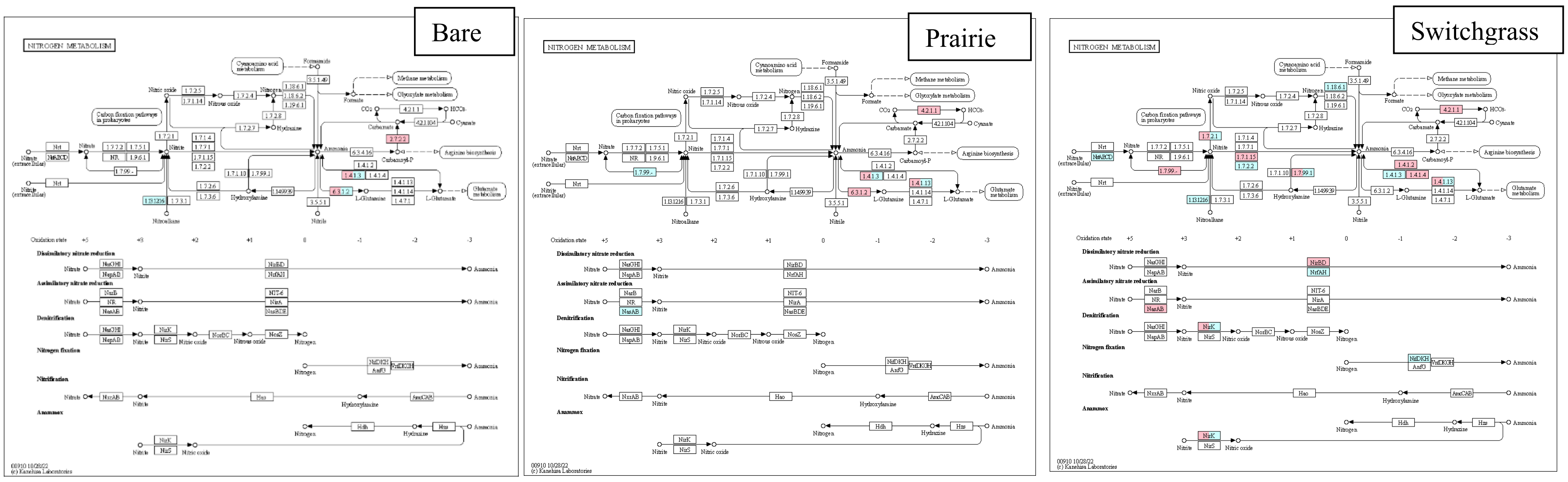
Bare
Prairie
Switchgrass
NITROGEN METABOLISM
NITROGEN METABOLISM
NITROGEN METABOLISM

b

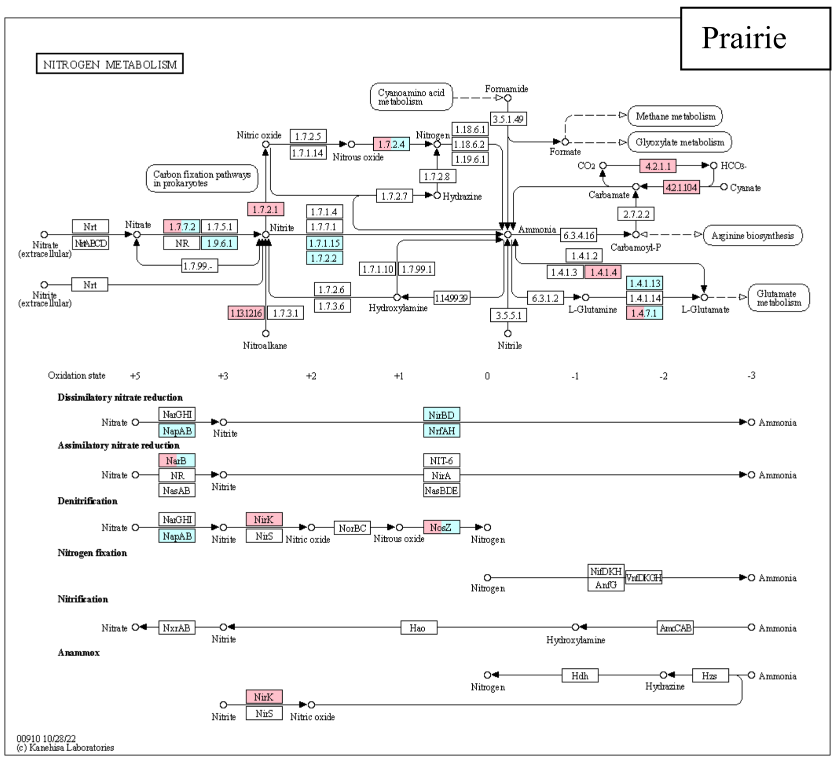

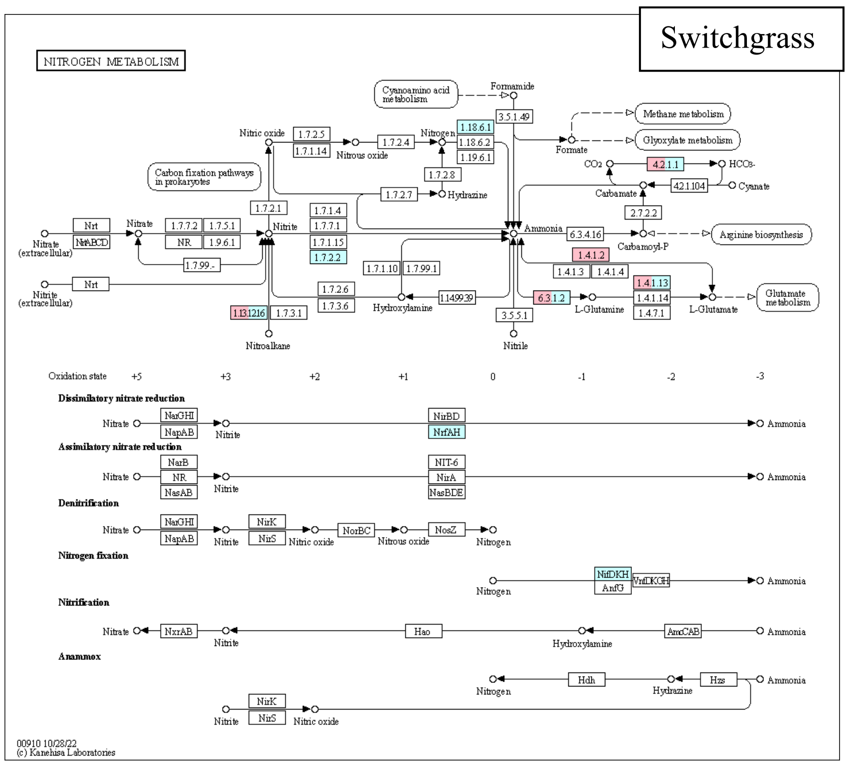

Figure S7. Genes enriched in the nitrogen metabolism in the studied plant systems at 24-hour (a) and 30-day incubations (b). Blue and pink mark enrichments in small and large pores, respectively. Diagrams shown with KEGG Copyright Permission 230979.

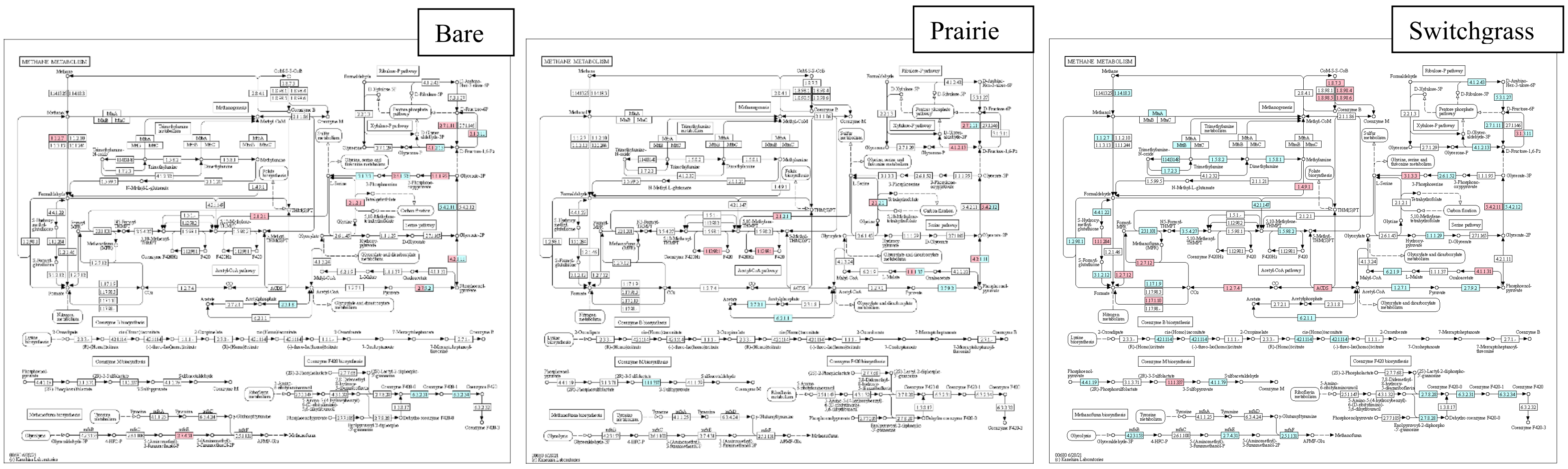

Figure S8. Genes enriched in the methane metabolism in the studied plant systems at 24-hour incubation. Blue and pink mark enrichments in small and large pores, respectively. Diagrams shown with KEGG Copyright Permission 230979.

Table S1. Schematic representation of the steps of pore filling procedure for adding glucose to intact soil cores of large or small pore experimental treatments.

		**Pores (Ø) filled**		
**Step**	**Matric potential**	**Small**	**Control**	**Large**
**1**	400 kPa	< 1 µm water	< 1 µm water	< 1 µm water
**2**	75 kPa	1-4 µm water	1-4 µm water	1-4 µm water
**3**	30 kPa	**4-10 µm glucose**	4-10 µm water	4-10 µm water
**4**	10 kPa	10-30 µm water	10-30 µm water	10-30 µm water
**5**	2 kPa	30-150 µm water	30-150 µm water	**30-150 µm glucose**

Table S2. Basic soil properties and pore characteristics of the bare soil, switchgrass, and prairie systems. Shown are means (first row) and standard errors (second row). Letters mark differences among the three vegetation systems for each soil characteristic that were significant at p<0.1(*italic*) or p<0.05 (**bold**). The statistical models for all variables included the fixed effect of the plant system and pore treatment, with their interaction, and the random effect of the field replication blocks. Since distances to pores were strongly correlated to the small pore volumes, comparisons among the plant systems and pore treatments were adjusted for the small pore volumes using ANCOVA.

**Soil property**	**Plant system**		
	Bare	Switchgrass	Prairie
Measures obtained from bulk soil and soil core (5 cm Ø) analyses			
Phosphorus, ppm	61.6**a**	14.6**b**	35.0**a**
	10.3	9.1	8.8
Potassium, ppm	113.0	90.0	139.8
	44.6	25.7	22.3
pH	5.0*c*	5.5*b*	5.8*a*
	0.2	0.1	0.1
CEC	6.3	6.5	7.6
	0.8	0.4	0.4
Soil organic C, %	1.04*b*	0.95*b*	1.51*a*
	0.10	0.12	0.13
Soil total N, %	0.097*b*	0.092*b*	0.130*a*
	0.007	0.009	0.010
Microbial biomass C, mg C kg^{-1} soil	120**a**	170**a**	382**b**
	46	41	42
Stone content, g g^{-1} soil	NA	0.054	0.025
		0.016	0.015
Root content, g g^{-1} soil	NA	0.0012	0.0022
		0.0006	0.0007
Soil bulk density, g cm^{-3}	NA	1.69**a**	1.47**b**
		0.05	0.05
Total porosity, %	NA	34.9**a**	43.5**b**
		2.1	2.1
Measures obtained from X-ray µCT image analyses of mini-cores (8 mm Ø)			
Image-based porosity (>4 µm Ø), %*	9.9	11.8	10.7
	1.5	1.5	1.6
Sub-resolution porosity (<4 µm Ø), %	NA	23.1	33.0
		3.2	3.2
Small pores (4-10 µm Ø), %	1.6	1.5	1.9
	0.17	0.18	0.20
Large pores (30-150 µm Ø), %	3.8	5.3	4.0
	0.6	0.6	0.6
Distance to >4 µm Ø pores, µm	34.9*ab*	37.2*a*	30.2*b*
	2.9	2.9	3.0
Particulate organic matter (POM), %	0.4**a**	1.9**b**	2.2**b**
	0.21	0.19	0.21
Distance to POM, µm	403*b*	300*b*	156*a*
	46	40	46
Large pores within active detritusphere, % of total large pores	62.7**a**	70.9**a**	95.3**b**
	6.1	5.3	6.1
Small pores within active detritusphere, % of total small pores	36.2**a**	50.5**a**	86.4**b**
	7.4	7.3	8.4

* Percent values in this portion of the table are expressed per total soil volume.

Table S3. ANOVA results for: 1) the ^{13}C-CO_2 emitted during the incubation as atom-% ^{13}C of emitted CO_2 and as % of the total applied ^{13}C, 2) the ^{13}C remaining in the soil after the incubation as mg/kg soil and as % of the total applied ^{13}C, 3) percent of the total ^{13}C remaining in the soil as dissolved organic C, and 4) as cytosol C. Shown are numerator (NumDF) and denominator (DenDF) degrees of freedom and p-values (**bold** when significant at 0.05, and ***italic*** when significant at 0.1 level). Because of substantial differences in variances of the 24 hr and 30 day data of the dissolved organic ^{13}C and cytosol ^{13}C, as well as significant interactions with the incubation time, the data analyses for these two variables were conducted separately for each incubation time point. The statistical models reflected the hierarchical structure of the analyses and included random effects of (i) experimental plots nested within vegetation systems for all studied variables and also (ii) soil cores nested within plots, vegetation systems and pore treatments for gas measurements and soil ^{13}C. To account for lack of normality, the soil ^{13}C were log-transformed.

	**CO_2 ^{13}C**				**Soil ^{13}C**			
**Effect**	**Degrees of freedom NumDF**	**DenDF**	**Atom-% ^{13}C**	**% applied**	**NumDF**	**DenDF**	**mg/kg soil**	**% applied**
System	1	4	0.35	0.60	1	4	0.34	0.13
Pores	1	28	0.69	0.93	1	4	**0.032**	***0.077***
Incubation time	3	28	**<.0001**	**0.0003**	1	8	**0.0001**	**0.0001**
System:Pores	1	28	0.27	***0.073***	1	4	0.60	0.46
System:Time	3	28	**0.007**	***0.079***	1	8	0.52	0.56
Pores:Time	3	28	0.55	0.98	1	8	0.22	0.18
System:Pores:Time	3	28	0.97	0.49	1	8	0.24	0.15

**Effect**	**Degrees of freedom**		**Dissolved organic ^{13}C, % of soil ^{13}C**		**Cytosol ^{13}C, % of soil ^{13}C**	
	**NumDF**	**DenDF**	**24hr**	**30day**	**24hr**	**30day**
System	1	4	***0.085***	0.27	0.85	**0.02**
Pores	1	4	0.93	0.16	0.68	0.24
System:Pores	1	4	0.57	***0.055***	0.69	0.89

Table S4. ANOVA results (p-values) for the diversity indexes at 24 hr incubations (comparing all three vegetation systems) and with time as an additional factor (comparing prairie and switchgrass systems only). Note that because of COVID19 lab closures bare soil samples were not subjected to the 30-day incubation. Therefore, two analyses were conducted: the first analysis was for just 24 hr data, which included all three soils and enabled comparisons between the bare soil and the other two system; the second analysis was conducted for combined 24 hr and 30 day data of switchgrass and prairie systems and enabled assessment of the time effect in these two systems. The data from all indexes were analyzed simultaneously with index as an additional factor. The statistical model reflected the hierarchical structure of the analyses and included random effects of (i) experimental plots nested within vegetation systems, (ii) soil cores nested within plots, vegetation systems and pore treatments, and (iii) soil core halves used for analyses at two incubation times nested in addition to previously listed terms also in incubation time. The statistical models were simplified by sequentially deleting 3rd order interactions that were not statistically significant at $p<0.3$. To account for a lack of normality, the indexes were log-transformed. Heterogeneity of variances among different indexes was accounted for by unequal variance analyses with Kenward-Rogers degrees of freedom adjustment (Milliken and Johnson 2009).

**Bare, Prairie and Switchgrass systems after 24 hr incubation**				**Prairie and Switchgrass systems after 24 hr and 30 day incubations**			
	**Degrees of freedom**				**Degrees of freedom**		
**Effect**	**numerator**	**denominator**	**p-value**	**Effect**	**numerator**	**denominator**	**p-value**
System	2	131	**<.0001**	System	1	158	**<0.01**
Index	5	92.4	**<.0001**	Index	5	119	**<.0001**
System * Index	10	116	**<.001**	Pores	2	158	**0.02**
Pores	2	130	0.96	Incubation time	1	158	0.15
System *Pores	4	130	0.62	System * Index	5	119	**0.04**
Pores* Index	10	116	0.99	System*Pores	2	158	0.24
				System*Time	1	50.3	0.34
				Pores* Index	10	150	0.29
				Index*Time	5	119	0.60

Table S5. Enriched taxa (Wilcoxon test, one sided, $p < 0.05$) present in both large and small pores of the soils of the three studied vegetation systems incubated for 24 hours.

Glucose added to large pores						Glucose added to small pores					
Bare soil		Switchgrass		Prairie		Bare soil		Switchgrass		Prairie	
Group	Enrichment level	Group	Enrichment level	Group	Enrichment level	Group	Enrichment level	Group	Enrichment level	Group	Enrichment level
Pseudomonas	10.99	*Pseudomonas*	10.19	*Pseudomonas*	21.39	*Planococcus99*	10.85	*Clostridium*	6.91	*Pseudomonas*	9.54
Planococcus99	9.25	*Burkholderia-Caballeronia-Paraburkholderia*	9.45	*Pseudarthrobacter97*	4.13	*Pseudomonas*	4.99	*Pseudomonas*	6.64	*Pseudarthrobacter97*	4.83
Micrococcaceae	4.90	*Duganella54*	3.50	*Clostridium*	3.26	*Micrococcaceae*	3.75	*Geobacter*	2.94	*Clostridium*	2.00
Burkholderia-Caballeronia-Paraburkholderia	2.64	*Burkholderiaceae*	2.88	*Burkholderiaceae*	1.91	*Burkholderia-Caballeronia-Paraburkholderia*	2.75	*Duganella54*	2.30	*Clostridium*	1.27
Planococcaceae	0.96	*Cupriavidus*	2.13	*Allorhizobium-Neorhizobium-Pararhizobium-Rhizobium*	0.99	*Planococcaceae*	2.40	*Pseudarthrobacter95*	1.54	*Burkholderiaceae*	0.78
uncultured	0.76	*Azoarcus98*	1.45	*Burkholderiaceae*	0.96	*Burkholderia-Caballeronia-Paraburkholderia*	1.37	*Burkholderia-Caballeronia-Paraburkholderia*	1.36	*Allorhizobium-Neorhizobium-Pararhizobium-Rhizobium*	0.77
Mucilaginibacter	0.76	*Clostridium*	1.34	*Burkholderiaceae*	0.60	*Paenibacillus98*	0.78	*Geobacter*	1.17	*Massilia9*	0.55
Paenibacillus98	0.54	*Massilia82*	1.24	*Pseudomonas*	0.42	*uncultured*	0.76	*Massilia82*	0.98	*Geobacter*	0.42
Burkholderiaceae	0.53	*Pseudarthrobacter95*	1.12	*Massilia9*	0.36	*Kitasatospora92*	0.49	*Clostridium*	0.67	*Geobacter*	0.32
uncultured	0.41	*Rhodocyclaceae*	1.04	*Clostridium*	0.29	*Massilia72*	0.47	*Burkholderiaceae*	0.66	*Yersinia96*	0.31
Rhodoferax85	0.40	*Burkholderiaceae*	0.78	*Burkholderia-Caballeronia-Paraburkholderia99*	0.22	*Mucilaginibacter*	0.42	*Burkholderiaceae*	0.66	*Clostridiaceae*	0.25
Mucilaginibacter	0.39	*Paenibacillus98*	0.73	*Clostridium*	0.17	*Burkholderia-Caballeronia-Paraburkholderia99*	0.37	*Cupriavidus*	0.59	*Rhizobiaceae*	0.20

Mesorhizobium74	0.38	*Burkholderia-Caballeronia-Paraburkholderia99*	0.70	*Burkholderia-Caballeronia-Paraburkholderia*	0.17	*Mucilaginibacter*	0.36	*Clostridium*	0.54	*Clostridium*	0.16
Curvibacter99	0.37	*Allorhizobium-Neorhizobium-Pararhizobium-Rhizobium*	0.57	*Rhizobiaceae*	0.17	*uncultured*	0.32	*Rhodocyclaceae*	0.53	*Burkholderiaceae*	0.12
Burkholderia-Caballeronia-Paraburkholderia	0.34	*Burkholderiaceae*	0.47	*Burkholderiaceae*	0.17	*Sphingomonas*	0.31	*Burkholderiaceae*	0.49	*Enterobacteriaceae*	0.11
Allorhizobium-Neorhizobium-Pararhizobium-Rhizobium99	0.27	*Burkholderiaceae*	0.40	*Pseudomonas*	0.16	*Burkholderiaceae*	0.29	*Burkholderiaceae*	0.41	*Flavobacterium*	0.10
Burkholderiaceae	0.27	*Burkholderiaceae*	0.40	*Burkholderiaceae*	0.12	*Massilia*	0.26	*Allorhizobium-Neorhizobium-Pararhizobium-Rhizobium*	0.35	*Pseudomonas*	0.09
Burkholderiaceae	0.19	*Burkholderiaceae*	0.39	*Burkholderiaceae*	0.12	*Curvibacter99*	0.24	*Clostridium*	0.35	*Paenibacillus*	0.08
Massilia	0.18	*Mucilaginibacter*	0.37	*Deltaproteobacteria*	0.12	*Mesorhizobium74*	0.24	*Burkholderiaceae*	0.28	*Paenibacillus99*	0.08
Sphingomonas94	0.18	*Clostridium*	0.33	*Saccharimonadales*	0.11	*Burkholderia-Caballeronia-Paraburkholderia*	0.19	*Burkholderiaceae*	0.25	*Deltaproteobacteria*	0.08
Sphingomonas	0.16	*Mucilaginibacter54*	0.29	*Burkholderia-Caballeronia-Paraburkholderia99*	0.09	*Allorhizobium-Neorhizobium-Pararhizobium-Rhizobium99*	0.17	*Paenibacillus99*	0.24	*Paenibacillus*	0.07
Burkholderiaceae	0.16	*Burkholderia-Caballeronia-Paraburkholderia*	0.25	*Yersinia96*	0.09	*Paenibacillus*	0.17	*Burkholderia-Caballeronia-Paraburkholderia97*	0.21	*uncultured*	0.07
Massilia97	0.15	*Paenibacillus*	0.19	*Paenibacillus*	0.08	*Rhodopseudomonas*	0.16	*Burkholderiaceae*	0.19	*Pseudomonas*	0.07
Burkholderiaceae	0.14	*Pseudomonas*	0.16	*Clostridium*	0.07	*Burkholderiaceae*	0.14	*Azoarcus98*	0.18	*Paenibacillus*	0.06
Paenibacillus	0.13	*Pseudomonas99*	0.16	*Paenibacillus99*	0.06	*Burkholderiaceae*	0.13	*Clostridium*	0.14	*Geobacter*	0.06
Kitasatospora92	0.11	*Burkholderiaceae*	0.15	*Paenibacillaceae*	0.06	*Burkholderiaceae*	0.11	*Clostridiaceae*	0.13	*Novosphingobium9*	0.05
Spirochaeta	0.10	*Burkholderia-Caballeronia-Paraburkholderia97*	0.12	*uncultured62*	0.04	*Variovorax84*	0.11	*Yersinia92*	0.12	*Sphingomonas95*	0.05

Rhizobiaceae	0.09	*Sphingobacteriaceae*	0.11	*Burkholderiaceae*	0.03	*Sphingomonas94*	0.08	*Pseudomonas99*	0.11	*Burkholderiaceae*	0.05
Massilia	0.08	*Allorhizobium-Neorhizobium-Pararhizobium-Rhizobium52*	0.09	*Pelosinus*	0.03	*Terrabacter*	0.08	*uncultured*	0.11	*Bacillales*	0.04
Paenibacillus	0.07	*Novosphingobium*	0.09	*Clostridium*	0.03	*Massilia97*	0.08	*Pelosinus*	0.10	*Paenibacillus99*	0.04
Pseudomonas	0.06	*Cupriavidus*	0.09	*Novosphingobium96*	0.02	*uncultured*	0.07	*Bacillaceae*	0.10	*Pseudomonas*	0.04
Terrimicrobium	0.05	*Novosphingobium99*	0.08	*Bacillales*	0.02	*Paenibacillus99*	0.06	*Novosphingobium98*	0.10	*Rhizobiales*	0.03
Flavobacterium	0.05	*Mucilaginibacter*	0.08	*Saccharimonadales*	0.02	*Mucilaginibacter99*	0.06	*Burkholderia-Caballeronia-Paraburkholderia*	0.10	*Novosphingobium97*	0.03
Asticcacaulis	0.05	*uncultured*	0.07	*Paenibacillus92*	0.02	*Ralstonia94*	0.05	*Burkholderia-Caballeronia-Paraburkholderia99*	0.09	*Saccharimonadales*	0.03
Sphingomonadaceae	0.05	*Janthinobacterium96*	0.05	*Enterobacteriaceae*	0.02	*uncultured99*	0.05	*Anaerosinus*	0.08	*Novosphingobium96*	0.03
uncultured89	0.04	*uncultured99*	0.05	*Pseudomonas*	0.02	*Paenarthrobacter55*	0.05	*Paenibacillus98*	0.08	*Clostridium*	0.02
Gammaproteobacteria	0.04	*Novosphingobium98*	0.04	*Flavobacterium*	0.02	*Pseudomonas98*	0.05	*Allorhizobium-Neorhizobium-Pararhizobium-Rhizobium52*	0.08	*Geobacter*	0.02
uncultured	0.04	*Clostridiaceae*	0.04	*Geobacter*	0.01	*Burkholderiaceae*	0.04	*Paenibacillus*	0.06	*Clostridium*	0.02
Paenibacillus	0.04	*Paenibacillus99*	0.04	*Clostridium*	0.01	*Massilia*	0.04	*Mucilaginibacter54*	0.04	*Clostridium*	0.02
Caulobacter98	0.04	*uncultured67*	0.04	*Clostridium*	0.01	*Flavobacterium*	0.04	*Mucilaginibacter*	0.03	*Burkholderia-Caballeronia-Paraburkholderia99*	0.02
Ralstonia94	0.04	*uncultured*	0.03	*Sphingomonas*	0.01	*Rhizobiaceae*	0.04	*Sphingobacteriaceae*	0.03	*Pelosinus*	0.02
Burkholderiaceae	0.04	*Paenibacillus99*	0.03			*Gammaproteobacteria*	0.04	*uncultured99*	0.03	*Flavobacterium*	0.01
Herminiimonas94	0.03	*Paenibacillus*	0.03			*Planococcaceae*	0.04	*Clostridium*	0.03	*Allorhizobium-Neorhizobium-Pararhizobium-Rhizobium54*	0.01
Rhodopseudomonas97	0.03	*Paenibacillus*	0.03			*Burkholderiaceae*	0.03	*Bacillales*	0.02	*Paenibacillus98*	0.01

Pseudomonas98	0.03	*Bacillales*	0.03			*Sphingomonadaceae*	0.03	*Clostridium*	0.02	*Burkholderiaceae*	0.01
Aminobacter59	0.03	*Duganella7*	0.03			*Asticcacaulis*	0.03	*Bacillales*	0.02		
Mucilaginibacter	0.03	*Pelosinus*	0.02			*Terrimicrobium*	0.03	*Janthinobacterium96*	0.02		
uncultured	0.03	*Yersinia92*	0.02			*Pseudomonas*	0.03	*Clostridium*	0.02		
Variovorax76	0.03	*uncultured99*	0.02			*Paenibacillaceae*	0.03	*Geobacter*	0.02		
Mucilaginibacter63	0.02	*Bacillaceae*	0.02			*Ammoniphilus99*	0.03	*Cupriavidus*	0.01		
Asticcacaulis	0.02	*uncultured*	0.02			*Labrys*	0.02	*Paenibacillus98*	0.01		
Spirochaeta	0.02	*Burkholderia-Caballeronia-Paraburkholderia*	0.02			*Caulobacter98*	0.02	*Duganella7*	0.01		
Labrys	0.02	*Proteobacteria*	0.02			*Niastella*	0.02	*Paenibacillus9*	0.01		
Asticcacaulis	0.02	*Anaerosinus*	0.01			*Fulvimonas5*	0.02	*Clostridiaceae*	0.01		
Pedobacter94	0.02	*Geobacter96*	0.01			*uncultured7*	0.02	*Novosphingobium98*	0.01		
Mucilaginibacter	0.02	*Clostridium*	0.01			*Rhodopseudomonas97*	0.02	*uncultured*	0.01		
Paenibacillaceae	0.02	*Paenibacillaceae*	0.01			*Spirochaeta*	0.02	*Proteobacteria*	0.01		
Niastella	0.02	*Bacillales*	0.01			*Paenibacillus*	0.02				
Terrabacter	0.02					*Mucilaginibacter63*	0.02				
Fulvimonas5	0.01					*Paenibacillus96*	0.02				
Planococcaceae	0.01					*Variovorax76*	0.02				
Burkholderiaceae	0.01					*Pedobacter94*	0.01				
Paenarthrobacter55	0.01					*Labrys*	0.01				
Dongia	0.01					*Asticcacaulis*	0.01				
						Herminiimonas94	0.01				
						Paenibacillus99	0.01				
						uncultured	0.01				
						Dongia	0.01				

Table S6. Enriched taxa (Wilcoxon test, one sided, $p < 0.05$) present in both large and small pores of the soils of switchgrass and prairie systems after 30-day incubation.

Glucose added to large pores				Glucose added to small pores			
Switchgrass		Prairie		Switchgrass		Prairie	
Group	Enrichment level	Group	Enrichment level	Group	Enrichment level	Group	Enrichment level
Burkholderia-Caballeronia-Paraburkholderia	13.56	*Pseudomonas*	2.57	*Burkholderiaceae*	0.83	*Nakamurella*	4.40
Pseudomonas	3.38	*Nakamurella*	2.13	*Burkholderiaceae*	0.51	*CandidatusXiphinematobacter*	1.81
Cupriavidus	2.49	*Mycobacterium*	1.11	*Mycobacterium*	0.31	*Burkholderiaceae*	1.75
Nakamurella	1.39	*Spirochaeta*	0.99	*Burkholderiaceae*	0.22	*Mycobacterium*	1.46
Burkholderiaceae	1.38	*Bradyrhizobium95*	0.90	*Nakamurella*	0.20	*Solirubrobacter*	1.45
Mycobacterium	1.15	*Burkholderiaceae*	0.77	*Burkholderiaceae*	0.16	*Spirochaeta*	1.45
Burkholderiaceae	0.93	*Solirubrobacter*	0.73	*uncultured*	0.11	*Intrasporangiaceae*	1.31
Duganella66	0.77	*Intrasporangiaceae*	0.68	*Microbacteriaceae*	0.10	*Burkholderiaceae*	1.10
Burkholderiaceae	0.77	*Bdellovibrio*	0.63	*Burkholderiaceae*	0.08	*Bacteria*	1.08
Azoarcus96	0.75	*Burkholderiaceae*	0.60	*Pseudomonas*	0.07	*Burkholderiaceae*	1.04
Spirochaeta	0.58	*CandidatusXiphinematobacter*	0.53	*Duganella66*	0.07	*mle-27*	1.02
Burkholderia-Caballeronia-Paraburkholderia96	0.52	*Burkholderiaceae*	0.45	*Burkholderia-Caballeronia-Paraburkholderia*	0.07	*Burkholderiaceae*	0.97
Allorhizobium-Neorhizobium-Pararhizobium-Rhizobium	0.44	*Variovorax6*	0.45	*Burkholderiaceae*	0.06	*Pseudomonas*	0.83
uncultured	0.44	*Microscillaceae*	0.41	*Massilia9*	0.05	*Solirubrobacter*	0.82
Chthoniobacter	0.31	*Burkholderiaceae*	0.33	*Chthoniobacter*	0.05	*Variovorax6*	0.74
Burkholderiaceae	0.26	*SBR3*	0.33	*Spirochaeta*	0.05	*SBR3*	0.65
Burkholderiaceae	0.25	*Agromyces98*	0.31	*Burkholderiaceae*	0.05	*uncultured*	0.57
Burkholderiaceae	0.25	*Bacteria*	0.30	*Burkholderia-Caballeronia-Paraburkholderia96*	0.04	*Acidibacter*	0.51
Burkholderia-Caballeronia-Paraburkholderia99	0.23	*Nakamurella*	0.30	*Chthoniobacter*	0.04	*Microscillaceae*	0.51

Cupriavidus	0.22	*Jatrophihabitans*	0.29	*Clostridium*	0.03	*Jatrophihabitans*	0.50
Microbacteriaceae	0.20	*uncultured*	0.28	*Spirochaeta*	0.03	*Nakamurella*	0.48
Massilia9	0.15	*Acidibacter*	0.26	*Nakamurella*	0.02	*Spirochaeta*	0.43
Burkholderiaceae	0.15	*Chthoniobacter*	0.26	*Rhizobiaceae*	0.02	*Spirochaeta*	0.41
vadinHA49	0.11	*Myxococcales*	0.26	*uncultured*	0.02	*Burkholderia-Caballeronia-Paraburkholderia98*	0.40
Polaromonas7	0.10	*Burkholderia-Caballeronia-Paraburkholderia98*	0.24	*Cytophaga*	0.02	*Agromyces98*	0.39
Saccharimonadales	0.09	*uncultured99*	0.23	*Spirochaeta*	0.02	*Bradyrhizobium95*	0.37
uncultured	0.08	*SBR3*	0.21	*Allorhizobium-Neorhizobium-Pararhizobium-Rhizobium*	0.02	*Nakamurella*	0.34
Clostridium	0.07	*Allorhizobium-Neorhizobium-Pararhizobium-Rhizobium99*	0.20	*Nakamurella*	0.02	*Marmoricola*	0.31
Spirochaeta	0.05	*Marmoricola*	0.17	*Azoarcus96*	0.02	*Mycobacterium*	0.31
Rhizobiaceae	0.04	*Pajaroellobacter*	0.16	*Chthoniobacter*	0.01	*Marmoricola98*	0.31
Cupriavidus98	0.04	*Nakamurella*	0.16	*Polaromonas7*	0.01	*BIrii4*	0.30
Cytophaga	0.03	*uncultured*	0.16	*Chthoniobacter*	0.01	*uncultured99*	0.30
uncultured	0.02	*Burkholderiaceae*	0.16	*Burkholderia-Caballeronia-Paraburkholderia99*	0.01	*Nocardioides98*	0.28
Chthoniobacter	0.02	*Marmoricola98*	0.16	*vadinHA49*	0.01	*uncultured99*	0.27
uncultured82	0.02	*Burkholderiaceae*	0.15	*Rhizobiaceae*	0.01	*Nocardioides*	0.25
vadinHA49	0.02	*A2b*	0.14	*Cupriavidus*	0.01	*Chthoniobacter*	0.25
uncultured	0.02	*SC-I-84*	0.13	*uncultured76*	0.01	*Ellin667*	0.24
Burkholderia-Caballeronia-Paraburkholderia	0.02	*Sulfurifustis*	0.13	*Mycobacterium*	0.01	*uncultured*	0.24
Cytophagaceae	0.02	*Spirochaeta*	0.13	*Saccharimonadales*	0.01	*Solirubrobacter*	0.23
Saccharimonadales	0.01	*Solirubrobacter*	0.13	*uncultured*	0.01	*Sulfurifustis*	0.23
Chthoniobacteraceae	0.01	*Terrabacter*	0.12			*SBR3*	0.23
Chthoniobacter	0.01	*Saccharimonadales*	0.12			*Saccharimonadales*	0.22
Burkholderiaceae	0.01	*Rhizobiaceae*	0.12			*Allorhizobium-Neorhizobium-Pararhizobium-Rhizobium99*	0.21
		uncultured	0.12			*Skermanella*	0.20

	Mycobacterium	0.12		*Myxococcales*	0.20
	uncultured	0.12		*Rhodococcus64*	0.19
	Burkholderia-Caballeronia-Paraburkholderia	0.12		*Terrabacter*	0.19
	Psychroglaciecola94	0.12		*Cytophagales*	0.17
	Burkholderiaceae	0.11		*A2b*	0.17
	Labrys	0.11		*Polyangiaceae*	0.17
	vadinHA49	0.11		*Psychroglaciecola94*	0.17
	Geobacter	0.10		*SBR3*	0.16
	SC-I-84	0.10		*vadinHA49*	0.16
	Skermanella	0.10		*Rhizobiaceae*	0.15
	Acidibacter	0.10		*Burkholderiaceae*	0.15
	Burkholderiaceae	0.09		*Pajaroellobacter*	0.15
	A2b	0.09		*Nocardioides*	0.15
	Rhizobiaceae	0.09		*Myxococcales*	0.14
	Cytophagales	0.08		*Burkholderiaceae*	0.13
	Salinispira	0.08		*Nocardiaceae*	0.13
	Chthoniobacter	0.08		*Rhizobiaceae*	0.13
	Burkholderiaceae	0.07		*Nocardioides*	0.12
	Bacteria	0.07		*Nocardioides*	0.12
	SBR3	0.07		*Mycobacterium*	0.12
	Devosia94	0.07		*Lineage*	0.11
	Myxococcales	0.07		*possible*	0.11
	Lineage	0.07		*Mycobacterium*	0.11
	Bdellovibrio	0.06		*Devosia94*	0.11
	Kribbella	0.06		*Labrys*	0.11
	Nocardioides	0.06		*Roseiflexaceae*	0.11
	Rhodococcus64	0.06		*Burkholderiaceae*	0.11
	Mycobacterium	0.06		*CandidatusAlysiosphaera*	0.10

	Saccharimonadales	0.05		*Nocardioides*	0.09
	Rhodococcus99	0.05		*Rhodococcus99*	0.09
	Nocardioides	0.05		*uncultured*	0.09
	SBR3	0.05		*Acidibacter*	0.08
	Ellin667	0.05		*SBR3*	0.08
	Chthoniobacter76	0.04		*Saccharimonadales*	0.08
	possible	0.04		*Streptomycetaceae*	0.08
	Rhizobiaceae	0.04		*Burkholderiaceae*	0.08
	Chthoniobacter	0.04		*Kribbella*	0.08
	Marmoricola94	0.04		*Geobacter*	0.07
	Streptomycetaceae	0.04		*Rhodococcus*	0.07
	Mycobacterium	0.04		*Solirubrobacter*	0.07
	SBR3	0.04		*Ohtaekwangia*	0.07
	Roseiflexaceae	0.04		*uncultured*	0.07
	Burkholderia-Caballeronia-Paraburkholderia	0.04		*Ellin667*	0.06
	Nordella	0.04		*Bacteria*	0.06
	Chthoniobacteraceae	0.04		*Chthoniobacter*	0.06
	Saccharimonadales	0.04		*uncultured*	0.06
	vadinHA49	0.04		*Nocardioides*	0.06
	Myxococcales	0.04		*Mycobacterium*	0.06
	WD2	0.04		*Geobacter*	0.05
	SBR3	0.03		*Saccharimonadales*	0.05
	Saccharimonadales	0.03		*Micromonosporaceae*	0.05
	Burkholderiaceae	0.03		*Salinispira*	0.05
	Myxococcales	0.03		*Geobacter*	0.05
	Burkholderiaceae	0.03		*Chthoniobacter76*	0.05
	Jatrophihabitans	0.03		*Myxococcales*	0.05
	Chloroflexi	0.03		*Marmoricola94*	0.05

	Saccharimonadales	0.03		*Rhizobiaceae*	0.05
	WD2	0.03		*WD2*	0.05
	Chthoniobacter	0.03		*possible*	0.04
	Rhizobiaceae	0.03		*Burkholderia-Caballeronia-Paraburkholderia*	0.04
	SBR3	0.03		*Omnitrophicaeota*	0.04
	Rhodobacteraceae	0.02		*Saccharimonadales*	0.04
	SBR3	0.02		*Frankiales*	0.04
	mle-27	0.02		*Solirubrobacteraceae*	0.04
	Micromonosporaceae	0.02		*Myxococcales*	0.04
	Chthoniobacteraceae	0.02		*Chloroflexi*	0.04
	MVP-88	0.02		*Psychroglaciecola98*	0.04
	Chloroflexi	0.02		*Rhodobacteraceae*	0.04
	SC-I-84	0.02		*A2b*	0.04
	Ohtaekwangia	0.02		*Nocardioides*	0.04
	CandidatusOmnitrophus	0.02		*Burkholderiaceae*	0.04
	Omnitrophicaeota	0.02		*Rhizobiaceae*	0.04
	uncultured	0.02		*Pajaroellobacter*	0.04
	Bdellovibrio	0.02		*Haliangium*	0.04
	SBR3	0.02		*SBR3*	0.04
	Aminobacter55	0.02		*mle-27*	0.04
	Cytophagales	0.02		*Nocardioides*	0.04
	Frankiales	0.02		*Saccharimonadales*	0.03
	Chthoniobacter	0.02		*Patescibacteria*	0.03
	Haliangium	0.02		*Verrucomicrobiae*	0.03
	uncultured	0.02		*Burkholderia-Caballeronia-Paraburkholderia*	0.03
	Bdellovibrio	0.02		*Jatrophihabitans*	0.03
	Bacteria	0.02		*Rhodanobacteraceae*	0.03
	Acidisphaera97	0.02		*SBR3*	0.03

vadinHA49	0.02	*Acidisphaera97*	0.03
uncultured	0.01	*Ohtaekwangia*	0.03
Cytophaga	0.01	*SBR3*	0.03
Patescibacteria	0.01	*mle-27*	0.03
Skermanella	0.01	*Skermanella*	0.03
uncultured	0.01	*SBR3*	0.03
Pajaroellobacter99	0.01	*vadinHA49*	0.03
Jatrophihabitans99	0.01	*Sulfurifustis*	0.03
Chthoniobacter	0.01	*SC-I-84*	0.03
Lineage	0.01	*Saccharimonadales*	0.02
Myxococcales	0.01	*Microlunatus*	0.02
SBR3	0.01	*Cytophagales*	0.02
Bacteria	0.01	*vadinHA49*	0.02
uncultured	0.01	*uncultured*	0.02
Anaeromyxobacter	0.01	*Nocardioides98*	0.02
Microlunatus	0.01	*Saccharimonadales*	0.02
BIrii4	0.01	*Aminobacter55*	0.02
CandidatusLloydbacteria	0.01	*CandidatusOmnitrophus*	0.02
Chthoniobacter65	0.01	*Chthoniobacter*	0.02
Chthoniobacter	0.01	*Saccharimonadales*	0.02
Roseiflexaceae	0.01	*Rhodanobacteraceae*	0.02
Terriglobus	0.01	*SBR3*	0.02
Sulfurifustis	0.01	*Jatrophihabitans99*	0.02
SBR3	0.01	*Lineage*	0.02
Saccharimonadales	0.01	*vadinHA49*	0.02
SBR3	0.01	*uncultured*	0.02
SBR3	0.01	*CandidatusXiphinematobacter83*	0.02
Myxococcales	0.01	*vadinHA49*	0.02
Verrucomicrobiae	0.01	*Cytophaga*	0.02

Polyangiaceae	0.01	*Chthoniobacter*	0.02
Bacteria	0.01	*Micromonosporaceae*	0.02
Saccharimonadales	0.01	*Rhodobacteraceae*	0.02
Cytophaga	0.01	*Bdellovibrio*	0.02
uncultured95	0.01	*Nocardioides*	0.02
		Bacteria	0.02
		Frankiales	0.02
		Saccharimonadales	0.02
		Nordella	0.02
		Burkholderiaceae	0.02
		BIrii4	0.02
		Roseiflexaceae	0.02
		MVP-88	0.02
		Chthoniobacter	0.02
		possible	0.02
		Rhizobiales	0.02
		Anaeromyxobacter	0.01
		CandidatusAlysiosphaera	0.01
		uncultured	0.01
		Pajaroellobacter	0.01
		Sulfurifustis	0.01
		WD2	0.01
		Ellin66799	0.01
		Myxococcales	0.01
		Chthoniobacter	0.01
		Chthoniobacteraceae	0.01
		Nocardioides	0.01
		Saccharimonadales	0.01
		Rhodobacteraceae	0.01

		Bacteria	0.01
		Myxococcales	0.01
		vadinHA49	0.01
		Devosia8	0.01
		Cytophaga	0.01
		uncultured	0.01
		Haliangium	0.01
		SBR3	0.01

Table S7. Enriched taxa (Wilcoxon test, one sided, $p < 0.05$) found only in the large or only in the small pore treatments at least twice (either in two systems or in two incubation dates or both) during the experiment, along with the information on their Gram positive/negative status, aerobic status, and key processes functional groups they are involved in as per FAPROTAX database (when available).

	After 24 hr incubation			After 30 day incubation		Possibly aerobic?	Processes	Gram-/+
	Bare	Switchgrass	Prairie	Switchgrass	Prairie			
**Large pores**								
Cellvibrio	+	+	+	+		Yes	Nitrogen fixation<br>Cellulolysis<br>Aerobic chemoheterotrophy	G-
Bacteriovorax	+				+	Yes	Nitrate reduction (assimilatory or dissimilatory)<br>Predatory or exoparasitic<br>Predator of G-	G-
Chitinophaga	+	+				Yes	Chitinolysis<br>Cellulolysis<br>Aerobic chemoheterotrophy<br>Nitrate reduction<br>Ureolysis	G-
Pseudoduganella	+	+	+			Yes		G-
Sphingobium	+				+	Yes	Ligninolysis<br>Aromatic compound degradation	G-
Crenobacter		+		+		Yes		G-
Mesorhizobium		+		+		Yes	Nitrogen fixation<br>Aerobic chemoheterotrophy<br>Ureolysis	G-
Paenarthrobacter		+	+			Yes		G+
Prosthecobacter		+		+		Yes	Aerobic chemoheterotrophy	G-
**Small pores**								
Oryzihumus	+				+	Yes	Aerobic chemoheterotrophy	G+
Pedosphaeraceae		+	+					

Spirochaeta		+	+				No/Yes	Nitrate reduction (assimilatory or dissimilatory)<br>Cellulolysis<br>Fermentation (anaerobic)	G-
Cellulomonas			+		+	+	No/Yes	Cellulolysis<br>Xylanolysis<br>Fermentation (anaerobic)<br>Aerobic chemoheterotrophy<br>Iron respiration(anaerobic)<br>Nitrate reduction (assimilatory or dissimilatory)<br>Ureolysis	G+
Dechlorosoma			+		+	+	No/Yes	Nitrate denitrification<br>Aerobic chemoheterotrophy<br>Dark iron oxidation<br>Nitrate respiration (anaerobic)	G-
Kaistia			+			+	Yes	Aerobic chemoheterotrophy	G-
Luteolibacter			+			+	Yes	Rhizosphere	G-
Microbacteriaceae			+			+	Yes	Aerobic chemoheterotrophy<br>Nitrate reduction (assimilatory or dissimilatory)<br>Ureolysis	G-
Fodinicola					+	+	Yes	Aerobic chemoheterotrophy<br>Ureolysis	G+
Gemmatimonas					+	+	Yes	Aerobic chemoheterotrophy	G-

Table S8. KO numbers and gene descriptions for KEGG glycolysis gluconeogenesis pathway (PATHko00010).

Gene Name	Gene Description
K00114	exaA; alcohol dehydrogenase (cytochrome c) [EC:1.1.2.8]
K00121	frmA, ADH5, adhC; S-(hydroxymethyl) glutathione dehydrogenase / alcohol dehydrogenase [EC:1.1.1.284 1.1.1.1]
K00128	ALDH; aldehyde dehydrogenase (NAD+) [EC:1.2.1.3]
K00131	gapN; glyceraldehyde-3-phosphate dehydrogenase (NADP+) [EC:1.2.1.9]
K00134	GAPDH, gapA; glyceraldehyde 3-phosphate dehydrogenase [EC:1.2.1.12]
K00161	PDHA, pdhA; pyruvate dehydrogenase E1 component alpha subunit [EC:1.2.4.1]
K00162	PDHB, pdhB; pyruvate dehydrogenase E1 component beta subunit [EC:1.2.4.1]
K00171	porD; pyruvate ferredoxin oxidoreductase delta subunit [EC:1.2.7.1]
K00174	korA, oorA, oforA; 2-oxoglutarate/2-oxoacid ferredoxin oxidoreductase subunit alpha [EC:1.2.7.3 1.2.7.11]
K00175	korB, oorB, oforB; 2-oxoglutarate/2-oxoacid ferredoxin oxidoreductase subunit beta [EC:1.2.7.3 1.2.7.11]
K00382	DLD, lpd, pdhD; dihydrolipoamide dehydrogenase [EC:1.8.1.4]
K00627	DLAT, aceF, pdhC; pyruvate dehydrogenase E2 component (dihydrolipoamide acetyltransferase) [EC:2.3.1.12]
K00845	glk; glucokinase [EC:2.7.1.2]
K00850	pfkA, PFK; 6-phosphofructokinase 1 [EC:2.7.1.11]
K00873	PK, pyk; pyruvate kinase [EC:2.7.1.40]
K00886	ppgK; polyphosphate glucokinase [EC:2.7.1.63]
K00927	PGK, pgk; phosphoglycerate kinase [EC:2.7.2.3]
K01006	ppdK; pyruvate, orthophosphate dikinase [EC:2.7.9.1]
K01007	pps, ppsA; pyruvate, water dikinase [EC:2.7.9.2]
K01222	E3.2.1.86A, celF; 6-phospho-beta-glucosidase [EC:3.2.1.86]
K01223	E3.2.1.86B, bglA; 6-phospho-beta-glucosidase [EC:3.2.1.86]
K01596	E4.1.1.32, pckA, PCK; phosphoenolpyruvate carboxykinase (GTP) [EC:4.1.1.32]
K01610	E4.1.1.49, pckA; phosphoenolpyruvate carboxykinase (ATP) [EC:4.1.1.49]
K01624	FBA, fbaA; fructose-bisphosphate aldolase, class II [EC:4.1.2.13]
K01689	ENO, eno; enolase [EC:4.2.1.11]
K01785	galM, GALM; aldose 1-epimerase [EC:5.1.3.3]
K01792	E5.1.3.15; glucose-6-phosphate 1-epimerase [EC:5.1.3.15]
K01803	TPI, tpiA; triosephosphate isomerase (TIM) [EC:5.3.1.1]
K01810	GPI, pgi; glucose-6-phosphate isomerase [EC:5.3.1.9]
K01834	PGAM, gpmA; 2,3-bisphosphoglycerate-dependent phosphoglycerate mutase [EC:5.4.2.11]
K01835	pgm; phosphoglucomutase [EC:5.4.2.2]
K01895	ACSS1_2, acs; acetyl-CoA synthetase [EC:6.2.1.1]
K01905	acdA; acetate---CoA ligase (ADP-forming) subunit alpha [EC:6.2.1.13]
K02446	glpX; fructose-1,6-bisphosphatase II [EC:3.1.3.11]
K02777	crr; sugar PTS system EIIA component [EC:2.7.1.-]
K02791	malX; maltose/glucose PTS system EIICB component [EC:2.7.1.199 2.7.1.208]
K03841	FBP, fbp; fructose-1,6-bisphosphatase I [EC:3.1.3.11]
K04072	adhE; acetaldehyde dehydrogenase / alcohol dehydrogenase [EC:1.2.1.10 1.1.1.1]
K11532	glpX-SEBP; fructose-1,6-bisphosphatase II / sedoheptulose-1,7-bisphosphatase [EC:3.1.3.11 3.1.3.37]
K11645	fbaB; fructose-bisphosphate aldolase, class I [EC:4.1.2.13]
K13810	tal-pgi; transaldolase / glucose-6-phosphate isomerase [EC:2.2.1.2 5.3.1.9]
K13953	adhP; alcohol dehydrogenase, propanol-preferring [EC:1.1.1.1]
K13979	yahK; alcohol dehydrogenase (NADP+) [EC:1.1.1.2]
K15633	gpmI; 2,3-bisphosphoglycerate-independent phosphoglycerate mutase [EC:5.4.2.12]
K15634	gpmB; 2,3-bisphosphoglycerate-dependent phosphoglycerate mutase [EC:5.4.2.11]

K15635	apgM; 2,3-bisphosphoglycerate-independent phosphoglycerate mutase [EC:5.4.2.12]
K16370	pfkB; 6-phosphofructokinase 2 [EC:2.7.1.11]

Table S9. KO numbers and gene descriptions for the KEGG methane metabolism pathway (PATHko00680).

Gene Name	Gene Description
K00018	hprA; glycerate dehydrogenase [EC:1.1.1.29]
K00024	mdh; malate dehydrogenase [EC:1.1.1.37]
K00058	serA, PHGDH; D-3-phosphoglycerate dehydrogenase / 2-oxoglutarate reductase [EC:1.1.1.95 1.1.1.399]
K00121	frmA, ADH5, adhC; S-(hydroxymethyl)glutathione dehydrogenase / alcohol dehydrogenase [EC:1.1.1.284 1.1.1.1]
K00122	FDH; formate dehydrogenase [EC:1.17.1.9]
K00123	fdoG, fdhF, fdwA; formate dehydrogenase major subunit [EC:1.17.1.9]
K00124	fdoH, fdsB; formate dehydrogenase iron-sulfur subunit
K00126	fdsD; formate dehydrogenase subunit delta [EC:1.17.1.9]
K00127	fdoI, fdsG; formate dehydrogenase subunit gamma
K00169	porA; pyruvate ferredoxin oxidoreductase alpha subunit [EC:1.2.7.1]
K00170	porB; pyruvate ferredoxin oxidoreductase beta subunit [EC:1.2.7.1]
K00171	porD; pyruvate ferredoxin oxidoreductase delta subunit [EC:1.2.7.1]
K00172	porC, porG; pyruvate ferredoxin oxidoreductase gamma subunit [EC:1.2.7.1]
K00194	cdhD, acsD; acetyl-CoA decarbonylase/synthase, CODH/ACS complex subunit delta [EC:2.1.1.245]
K00196	cooF; anaerobic carbon-monoxide dehydrogenase iron sulfur subunit
K00197	cdhE, acsC; acetyl-CoA decarbonylase/synthase, CODH/ACS complex subunit gamma [EC:2.1.1.245]
K00198	cooS, acsA; anaerobic carbon-monoxide dehydrogenase catalytic subunit [EC:1.2.7.4]
K00200	fwdA, fmdA; formylmethanofuran dehydrogenase subunit A [EC:1.2.7.12]
K00201	fwdB, fmdB; formylmethanofuran dehydrogenase subunit B [EC:1.2.7.12]
K00202	fwdC, fmdC; formylmethanofuran dehydrogenase subunit C [EC:1.2.7.12]
K00317	dmd-tmd; dimethylamine/trimethylamine dehydrogenase [EC:1.5.8.1 1.5.8.2]
K00320	mer; 5,10-methylenetetrahydromethanopterin reductase [EC:1.5.98.2]
K00442	frhD; coenzyme F420 hydrogenase subunit delta
K00600	glyA, SHMT; glycine hydroxymethyltransferase [EC:2.1.2.1]
K00625	E2.3.1.8, pta; phosphate acetyltransferase [EC:2.3.1.8]
K00672	ftr; formylmethanofuran--tetrahydromethanopterin N-formyltransferase [EC:2.3.1.101]
K00830	AGXT; alanine-glyoxylate transaminase / serine-glyoxylate transaminase / serine-pyruvate transaminase [EC:2.6.1.44 2.6.1.45 2.6.1.51]
K00831	serC, PSAT1; phosphoserine aminotransferase [EC:2.6.1.52]
K00850	pfkA, PFK; 6-phosphofructokinase 1 [EC:2.7.1.11]
K00863	DAK, TKFC; triose/dihydroxyacetone kinase / FAD-AMP lyase (cyclizing) [EC:2.7.1.28 2.7.1.29 4.6.1.15]
K00918	pfkC; ADP-dependent phosphofructokinase/glucokinase [EC:2.7.1.146 2.7.1.147]
K00925	ackA; acetate kinase [EC:2.7.2.1]
K01007	pps, ppsA; pyruvate, water dikinase [EC:2.7.9.2]
K01070	frmB, ESD, fghA; S-formylglutathione hydrolase [EC:3.1.2.12]
K01079	serB, PSPH; phosphoserine phosphatase [EC:3.1.3.3]
K01086	fbp-SEBP; fructose-1,6-bisphosphatase I / sedoheptulose-1,7-bisphosphatase [EC:3.1.3.11 3.1.3.37]
K01499	mch; methenyltetrahydromethanopterin cyclohydrolase [EC:3.5.4.27]
K01595	ppc; phosphoenolpyruvate carboxylase [EC:4.1.1.31]
K01622	K01622; fructose 1,6-bisphosphate aldolase/phosphatase [EC:4.1.2.13 3.1.3.11]
K01623	ALDO; fructose-bisphosphate aldolase, class I [EC:4.1.2.13]
K01624	FBA, fbaA; fructose-bisphosphate aldolase, class II [EC:4.1.2.13]
K01689	ENO, eno; enolase [EC:4.2.1.11]
K01834	PGAM, gpmA; 2,3-bisphosphoglycerate-dependent phosphoglycerate mutase [EC:5.4.2.11]
K01895	ACSS1_2, acs; acetyl-CoA synthetase [EC:6.2.1.1]
K02203	thrH; phosphoserine / homoserine phosphotransferase [EC:3.1.3.3 2.7.1.39]
K02446	glpX; fructose-1,6-bisphosphatase II [EC:3.1.3.11]

K03388	hdrA2; heterodisulfide reductase subunit A2 [EC:1.8.7.3 1.8.98.4 1.8.98.5 1.8.98.6]
K03389	hdrB2; heterodisulfide reductase subunit B2 [EC:1.8.7.3 1.8.98.4 1.8.98.5 1.8.98.6]
K03390	hdrC2; heterodisulfide reductase subunit C2 [EC:1.8.7.3 1.8.98.4 1.8.98.5 1.8.98.6]
K03396	gfa; S-(hydroxymethyl)glutathione synthase [EC:4.4.1.22]
K03532	torC; trimethylamine-N-oxide reductase (cytochrome c), cytochrome c-type subunit TorC
K03533	torD; TorA specific chaperone
K03841	FBP, fbp; fructose-1,6-bisphosphatase I [EC:3.1.3.11]
K04041	fbp3; fructose-1,6-bisphosphatase III [EC:3.1.3.11]
K05299	fdhA; formate dehydrogenase (NADP+) alpha subunit [EC:1.17.1.10]
K05884	comC; L-2-hydroxycarboxylate dehydrogenase (NAD+) [EC:1.1.1.337]
K05979	comB; 2-phosphosulfolactate phosphatase [EC:3.1.3.71]
K06034	comD; sulfopyruvate decarboxylase subunit alpha [EC:4.1.1.79]
K06914	mfnD; tyramine---L-glutamate ligase [EC:6.3.4.24]
K07072	mfnF; (4-(4-[2-(gamma-L-glutamylamino)ethyl]phenoxymethyl)furan-2-yl)methanamine synthase [EC:2.5.1.131]
K07144	mfnE; 5-(aminomethyl)-3-furanmethanol phosphate kinase [EC:2.7.4.31]
K07811	torA; trimethylamine-N-oxide reductase (cytochrome c) [EC:1.7.2.3]
K07812	torZ; trimethylamine-N-oxide reductase (cytochrome c) [EC:1.7.2.3]
K08093	hxlA; 3-hexulose-6-phosphate synthase [EC:4.1.2.43]
K08094	hxlB; 6-phospho-3-hexuloisomerase [EC:5.3.1.27]
K08097	comA; phosphosulfolactate synthase [EC:4.4.1.19]
K08685	qhpA; quinohemoprotein amine dehydrogenase [EC:1.4.9.1]
K08691	mcl; malyl-CoA/(S)-citramalyl-CoA lyase [EC:4.1.3.24 4.1.3.25]
K08692	mtkB; malate-CoA ligase subunit alpha [EC:6.2.1.9]
K09733	mfnB; (5-formylfuran-3-yl)methyl phosphate synthase [EC:4.2.3.153]
K10713	fae; 5,6,7,8-tetrahydromethanopterin hydro-lyase [EC:4.2.1.147]
K10714	mtdB; methylene-tetrahydromethanopterin dehydrogenase [EC:1.5.1.-]
K10944	pmoA-amoA; methane/ammonia monooxygenase subunit A [EC:1.14.18.3 1.14.99.39]
K10945	pmoB-amoB; methane/ammonia monooxygenase subunit B
K10946	pmoC-amoC; methane/ammonia monooxygenase subunit C
K11212	cofD; LPPG:FO 2-phospho-L-lactate transferase [EC:2.7.8.28]
K11261	fwdE, fmdE; formylmethanofuran dehydrogenase subunit E [EC:1.2.7.12]
K11529	gck, gckA, GLYCTK; glycerate 2-kinase [EC:2.7.1.165]
K11532	glpX-SEBP; fructose-1,6-bisphosphatase II / sedoheptulose-1,7-bisphosphatase [EC:3.1.3.11 3.1.3.37]
K11645	fbaB; fructose-bisphosphate aldolase, class I [EC:4.1.2.13]
K11779	fbiC; FO synthase [EC:2.5.1.147 4.3.1.32]
K11780	cofG; 7,8-didemethyl-8-hydroxy-5-deazariboflavin synthase [EC:4.3.1.32]
K11781	cofH; 5-amino-6-(D-ribitylamino)uracil---L-tyrosine 4-hydroxyphenyl transferase [EC:2.5.1.147]
K12234	cofE; coenzyme F420-0:L-glutamate ligase / coenzyme F420-1:gamma-L-glutamate ligase [EC:6.3.2.31 6.3.2.34]
K13039	comE; sulfopyruvate decarboxylase subunit beta [EC:4.1.1.79]
K13788	pta; phosphate acetyltransferase [EC:2.3.1.8]
K13831	hps-phi; 3-hexulose-6-phosphate synthase / 6-phospho-3-hexuloisomerase [EC:4.1.2.43 5.3.1.27]
K14067	mtkA; malate-CoA ligase subunit beta [EC:6.2.1.9]
K14080	mtaA; [methyl-Co(III) methanol/glycine betaine-specific corrinoid protein]:coenzyme M methyltransferase [EC:2.1.1.246 2.1.1.377]
K14083	mttB; trimethylamine---corrinoid protein Co-methyltransferase [EC:2.1.1.250]
K14126	mvhA, vhuA, vhcA; F420-non-reducing hydrogenase large subunit [EC:1.12.99.- 1.8.98.5]
K14127	mvhD, vhuD, vhcD; F420-non-reducing hydrogenase iron-sulfur subunit [EC:1.12.99.- 1.8.98.5 1.8.98.6]
K14128	mvhG, vhuG, vhcG; F420-non-reducing hydrogenase small subunit [EC:1.12.99.- 1.8.98.5]
K14941	cofC, fbiD; 2-phospho-L-lactate/phosphoenolpyruvate guanylyltransferase [EC:2.7.7.68 2.7.7.105]
K15022	fdhB; formate dehydrogenase (NADP+) beta subunit [EC:1.17.1.10]
K15228	mauA; methylamine dehydrogenase light chain [EC:1.4.9.1]
K15229	mauB; methylamine dehydrogenase heavy chain [EC:1.4.9.1]

K15633	gpmI; 2,3-bisphosphoglycerate-independent phosphoglycerate mutase [EC:5.4.2.12]
K15634	gpmB; 2,3-bisphosphoglycerate-dependent phosphoglycerate mutase [EC:5.4.2.11]
K15635	apgM; 2,3-bisphosphoglycerate-independent phosphoglycerate mutase [EC:5.4.2.12]
K16254	mxaJ; mxaJ protein
K16256	mxaA; mxaA protein
K16257	mxaC; mxaC protein
K16258	mxaK; mxaK protein
K16259	mxaL; mxaL protein
K16260	mxaD; mxaD protein
K16306	K16306; fructose-bisphosphate aldolase / 2-amino-3,7-dideoxy-D-threo-hept-6-ulosonate synthase [EC:4.1.2.13 2.2.1.10]
K16370	pfkB; 6-phosphofructokinase 2 [EC:2.7.1.11]
K16792	aksD; methanogen homoaconitase large subunit [EC:4.2.1.114]
K16793	aksE; methanogen homoaconitase small subunit [EC:4.2.1.114]
K17067	mdo; formaldehyde dismutase / methanol dehydrogenase [EC:1.2.98.1 1.1.99.37]
K18277	tmm; trimethylamine monooxygenase [EC:1.14.13.148]

Table S10. KO numbers and gene descriptions for the KEGG nitrogen metabolism pathway (PATHko00680).

Gene Name	Gene Description
K00261	GLUD1_2, gdhA; glutamate dehydrogenase (NAD(P)+) [EC:1.4.1.3]
K00262	E1.4.1.4, gdhA; glutamate dehydrogenase (NADP+) [EC:1.4.1.4]
K00265	gltB; glutamate synthase (NADPH) large chain [EC:1.4.1.13]
K00266	gltD; glutamate synthase (NADPH) small chain [EC:1.4.1.13]
K00284	GLU, gltS; glutamate synthase (ferredoxin) [EC:1.4.7.1]
K00360	nasB; assimilatory nitrate reductase electron transfer subunit [EC:1.7.99.-]
K00362	nirB; nitrite reductase (NADH) large subunit [EC:1.7.1.15]
K00367	narB; ferredoxin-nitrate reductase [EC:1.7.7.2]
K00368	nirK; nitrite reductase (NO-forming) [EC:1.7.2.1]
K00372	nasA; assimilatory nitrate reductase catalytic subunit [EC:1.7.99.-]
K00376	nosZ; nitrous-oxide reductase [EC:1.7.2.4]
K00459	ncd2, npd; nitronate monooxygenase [EC:1.13.12.16]
K00926	arcC; carbamate kinase [EC:2.7.2.2]
K01673	cynT, can; carbonic anhydrase [EC:4.2.1.1]
K01674	cah; carbonic anhydrase [EC:4.2.1.1]
K01725	cynS; cyanate lyase [EC:4.2.1.104]
K01915	glnA, GLUL; glutamine synthetase [EC:6.3.1.2]
K02567	napA; nitrate reductase (cytochrome) [EC:1.9.6.1]
K02568	napB; nitrate reductase (cytochrome), electron transfer subunit
K02586	nifD; nitrogenase molybdenum-iron protein alpha chain [EC:1.18.6.1]
K02588	nifH; nitrogenase iron protein NifH
K02591	nifK; nitrogenase molybdenum-iron protein beta chain [EC:1.18.6.1]
K03385	nrfA; nitrite reductase (cytochrome c-552) [EC:1.7.2.2]
K05601	hcp; hydroxylamine reductase [EC:1.7.99.1]
K15371	GDH2; glutamate dehydrogenase [EC:1.4.1.2]
K15579	nrtD, cynD; nitrate/nitrite transport system ATP-binding protein
K15876	nrfH; cytochrome c nitrite reductase small subunit

Table S11. KO numbers and gene descriptions for the KEGG citrate cycle (TCA cycle) pathway (PATHko00020).

Gene Name	Gene Description
K00024	mdh; malate dehydrogenase [EC:1.1.1.37]
K00025	MDH1; malate dehydrogenase [EC:1.1.1.37]
K00030	IDH3; isocitrate dehydrogenase (NAD+) [EC:1.1.1.41]
K00031	IDH1, IDH2, icd; isocitrate dehydrogenase [EC:1.1.1.42]
K00116	mqo; malate dehydrogenase (quinone) [EC:1.1.5.4]
K00161	PDHA, pdhA; pyruvate dehydrogenase E1 component alpha subunit [EC:1.2.4.1]
K00162	PDHB, pdhB; pyruvate dehydrogenase E1 component beta subunit [EC:1.2.4.1]
K00163	aceE; pyruvate dehydrogenase E1 component [EC:1.2.4.1]
K00164	OGDH, sucA; 2-oxoglutarate dehydrogenase E1 component [EC:1.2.4.2]
K00171	porD; pyruvate ferredoxin oxidoreductase delta subunit [EC:1.2.7.1]
K00174	korA, oorA, oforA; 2-oxoglutarate/2-oxoacid ferredoxin oxidoreductase subunit alpha [EC:1.2.7.3 1.2.7.11]
K00175	korB, oorB, oforB; 2-oxoglutarate/2-oxoacid ferredoxin oxidoreductase subunit beta [EC:1.2.7.3 1.2.7.11]
K00176	korD, oorD; 2-oxoglutarate ferredoxin oxidoreductase subunit delta [EC:1.2.7.3]
K00177	korC, oorC; 2-oxoglutarate ferredoxin oxidoreductase subunit gamma [EC:1.2.7.3]
K00239	sdhA, frdA; succinate dehydrogenase / fumarate reductase, flavoprotein subunit [EC:1.3.5.1 1.3.5.4]
K00240	sdhB, frdB; succinate dehydrogenase / fumarate reductase, iron-sulfur subunit [EC:1.3.5.1 1.3.5.4]
K00241	sdhC, frdC; succinate dehydrogenase / fumarate reductase, cytochrome b subunit
K00245	frdB; fumarate reductase iron-sulfur subunit [EC:1.3.5.4]
K00246	frdC; fumarate reductase subunit C
K00247	frdD; fumarate reductase subunit D
K00382	DLD, lpd, pdhD; dihydrolipoamide dehydrogenase [EC:1.8.1.4]
K00627	DLAT, aceF, pdhC; pyruvate dehydrogenase E2 component (dihydrolipoamide acetyltransferase) [EC:2.3.1.12]
K00658	DLST, sucB; 2-oxoglutarate dehydrogenase E2 component (dihydrolipoamide succinyltransferase) [EC:2.3.1.61]
K01596	E4.1.1.32, pckA, PCK; phosphoenolpyruvate carboxykinase (GTP) [EC:4.1.1.32]
K01610	E4.1.1.49, pckA; phosphoenolpyruvate carboxykinase (ATP) [EC:4.1.1.49]
K01616	kgd; multifunctional 2-oxoglutarate metabolism enzyme [EC:2.2.1.5 4.1.1.71 1.2.4.2 2.3.1.61]
K01647	CS, gltA; citrate synthase [EC:2.3.3.1]
K01676	E4.2.1.2A, fumA, fumB; fumarate hydratase, class I [EC:4.2.1.2]
K01679	E4.2.1.2B, fumC, FH; fumarate hydratase, class II [EC:4.2.1.2]
K01681	ACO, acnA; aconitate hydratase [EC:4.2.1.3]
K01682	acnB; aconitate hydratase 2 / 2-methylisocitrate dehydratase [EC:4.2.1.3 4.2.1.99]
K01902	sucD; succinyl-CoA synthetase alpha subunit [EC:6.2.1.5]
K01903	sucC; succinyl-CoA synthetase beta subunit [EC:6.2.1.5]
K01958	PC, pyc; pyruvate carboxylase [EC:6.4.1.1]
K01960	pycB; pyruvate carboxylase subunit B [EC:6.4.1.1]

Table S12. Significantly enriched genes ($p < 0.05$) associated with the KEGG glycolysis gluconeogenesis pathway (X indicates the presence of the enriched genes).

Gene Name	Bare soil, 24 hr, large pores	Bare soil, 24 hr, small pores	High diversity prairie, 24 hr, large pores	High diversity prairie, 24 hr, small pores	High diversity prairie, 30 days, large pores	High diversity prairie, 30 days, small pores	Monoculture switchgrass, 24 hr, large pores	Monoculture switchgrass, 24 hr, small pores	Monoculture switchgrass, 30 days, large pores	Monoculture switchgrass, 30 days, small pores
K00114							X			X
K00121							X		X	X
K00128									X	X
K00131	X	X			X					X
K00134			X	X	X	X		X	X	X
K00161						X	X			X
K00162						X	X			X
K00171							X			
K00174			X	X	X	X		X		
K00175			X	X	X	X		X		
K00382		X							X	X
K00627						X	X			X
K00845			X	X	X	X		X		
K00850	X		X	X	X			X		
K00873	X		X	X				X	X	
K00886			X	X				X		X
K00927	X	X	X	X				X	X	
K01006	X	X	X	X			X	X	X	X
K01007		X		X				X		
K01222					X	X				
K01223	X									
K01596							X			
K01610	X	X			X					
K01624	X	X	X	X		X	X	X		
K01689	X	X	X	X				X	X	
K01785						X				
K01792					X		X			
K01803	X	X	X	X				X	X	
K01810	X	X	X	X				X	X	
K01834							X			X
K01835	X	X				X	X			
K01895					X	X				
K01905			X	X				X		
K02446	X	X			X	X				
K02777	X									
K02791	X				X					
K03841					X					
K04072										X
K11532							X		X	X
K11645			X						X	
K13810					X	X			X	
K13953		X			X	X				
K13979	X	X								
K15633	X		X	X	X			X	X	X
K15634		X								X
K15635			X	X				X		
K16370					X	X				X

Table S13. Significantly enriched genes ($p < 0.05$) associated with the KEGG methane metabolism pathway (X indicates the presence of the enriched genes).

Gene Name	Bare soil, 24 hr, large pores	Bare soil, 24 hr, small pores	High diversity prairie, 24 hr, large pores	High diversity prairie, 24 hr, small pores	High diversity prairie, 30 days, large pores	High diversity prairie, 30 days, small pores	Monoculture switchgrass, 24 hr, large pores	Monoculture switchgrass, 24 hr, small pores	Monoculture switchgrass, 30 days, large pores	Monoculture switchgrass, 30 days, small pores
K00018								X		X
K00024			X	X	X	X				
K00058	X				X	X				X
K00121						X	X			
K00122										X
K00123							X	X	X	
K00124							X	X	X	
K00126							X	X		
K00127								X		X
K00169								X		X
K00170								X		X
K00171							X			X
K00172								X		X
K00194							X			X
K00196								X		X
K00197							X			
K00198							X			X
K00200								X		X
K00201								X		X
K00202								X		X
K00317								X		X
K00320								X		X
K00442			X							
K00600	X		X	X		X				
K00625										X
K00672								X		X
K00830										X
K00831	X	X				X		X	X	
K00850	X		X	X	X	X				X
K00863										X
K00918										X
K00925				X					X	X
K01007	X	X		X	X			X		X
K01070								X	X	
K01079	X	X					X		X	
K01086								X		X
K01499								X		X
K01595					X	X	X		X	
K01622								X		
K01623								X		X
K01624	X	X	X	X	X	X	X			X
K01689	X	X	X	X	X				X	

K01834							X			
K01895				X	X	X		X	X	
K02203		X								X
K02446	X	X			X	X				
K03388							X			
K03389							X			X
K03390							X			X
K03396								X		X
K03532					X	X		X		X
K03533								X		X
K03841					X	X		X		X
K04041										X
K05299							X			X
K05884				X	X		X		X	
K05979					X	X				X
K06034								X		X
K06914										X
K07072								X		X
K07144	X							X		X
K07811								X		X
K07812								X		X
K08093								X		X
K08094								X		X
K08097					X	X		X		X
K08685							X			X
K08691										X
K08692								X		
K09733								X		X
K10713								X		X
K10714										X
K10944								X		X
K10945								X		X
K10946								X		X
K11212					X	X		X		
K11261							X			X
K11529										X
K11532							X	X	X	
K11645			X						X	
K11779					X	X				
K11780									X	
K11781									X	
K12234		X			X	X		X		
K13039								X		X
K13788		X			X	X				
K13831					X					X
K14067								X		
K14080								X		X
K14083								X		X
K14126										X
K14127						X				
K14128										X

K14941					X	X				
K15022										X
K15228							X			X
K15229							X			X
K15633			X	X	X			X	X	
K15634		X								
K15635			X	X						
K16254										X
K16256										X
K16257										X
K16258										X
K16259										X
K16260	X							X		
K16306								X		
K16370					X	X		X		
K16792								X		
K16793								X		
K17067								X		X
K18277								X		X

Table S14. Significantly enriched genes ($p < 0.05$) associated nitrogen metabolism (X indicates the presence of the enriched genes).

Gene Name		Bare soil, 24 hr, large pores	Bare soil, 24 hr, small pores	High diversity prairie, 24 hr, large pores	High diversity prairie, 24 hr, small pores	High diversity prairie, 30 days, large pores	High diversity prairie, 30 days, small pores	Monoculture switchgrass, 24 hr, large pores	Monoculture switchgrass, 24 hr, small pores	Monoculture switchgrass, 30 days, large pores	Monoculture switchgrass, 30 days, small pores
K00261	1.4.1.3	X	X	X	X				X		
K00262	1.4.1.4					X		X			
K00265	1.4.1.13			X	X		X	X	X	X	
K00266	1.4.1.13			X	X					X	X
K00284	1.4.7.1					X	X				
K00360	1.7.99				X				X		
K00362	1.7.1.12						X	X			
K00367	1.7.7.2					X	X				
K00368	1.7.2.1					X		X	X		
K00372	1.7.99							X			
K00376	1.7.2.4					X	X				
K00459	1.13.12.16		X			X			X	X	X
K00926	2.7.2.2	X									
K01673	4.2.1.1					X	X		X	X	X
K01674	4.2.1.1			X		X		X			
K01725	4.2.1.104					X					
K01915	6.3.1.2	X	X	X						X	X
K02567	1.9.6.1						X				
K02568							X				
K02586	1.18.6.1								X		X
K02588									X		
K02591	1.18.6.1								X		X
K03385	1.7.2.2.						X		X		X

K05601	1.7.99.1							X	X		
K15371	1.4.1.2							X		X	X
K15579									X		
K15876											X

Table S15. Significantly enriched genes ($p < 0.05$) associated with the KEGG citrate cycle (TCA cycle) (X indicates the presence of the enriched genes).

Gene Name		Bare soil, 24 hr, large pores	Bare soil, 24 hr, small pores	High diversity prairie, 24 hr, large pores	High diversity prairie, 24 hr, small pores	High diversity prairie, 30 days, large pores	High diversity prairie, 30 days, small pores	Monoculture switchgrass, 24 hr, large pores	Monoculture switchgrass, 24 hr, small pores	Monoculture switchgrass, 30 days, large pores	Monoculture switchgrass, 30 days, small pores
K00024	1.1.1.37	X		X	X				X		
K00025	1.1.1.37									X	
K00030	1.1.1.41			X							
K00031	1.1.1.42			X	X	X					
K00116	1.1.5.4		X			X	X				
K00161	1.2.4.1						X	X	X		X
K00162	1.2.4.1						X	X	X		X
K00163	1.2.4.1								X		
K00164	1.2.4.2	X	X			X	X			X	
K00171	1.2.7.1							X			
K00174	1.2.7.3 1.2.7.11			X	X	X	X				
K00175	1.2.7.3 1.2.7.11			X	X	X	X				
K00176	1.2.7.3							X	X		
K00177	1.2.7.3							X	X		
K00239	1.3.5.1. 1.3.5.4			X	X		X		X		
K00240	1.3.5.1. 1.3.5.4			X	X		X		X		
K00241				X	X					X	
K00245	1.3.5.4			X	X		X				
K00246				X	X		X				
K00247			X								X
K00382	1.8.1.4		X								X
K00627	2.3.1.12						X	X	X		X
K00658	2.3.1.61	X	X	X	X	X	X				X

K01596	4.1.1.32							X	X		
K01610	4.1.1.49	X	X			X	X				
K01616	2.2.1.5 etc							X	X		
K01647	2.3.3.1	X	X	X	X					X	X
K01676	4.2.1.2							X	X		X
K01679	4.2.1.2						X				
K01681	4.2.1.3	X	X	X	X						
K01682	4.2.1.3	X	X								
K01902	6.2.1.5	X	X	X	X					X	
K01903	6.2.1.5	X	X	X	X					X	
K01958	6.4.1.1.										X
K01960	6.4.1.1					X					
