# Supplementary material for: Composition and metabolism of microbial communities in soil pores
Source: Nat Commun. 2024 Apr 27;15:3578. doi: 10.1038/s41467-024-47755-x (PMC11055953; doi:10.1038/s41467-024-47755-x)
Supplement: Supplementary file 3 — Reporting Summary [file 41467_2024_47755_MOESM3_ESM.pdf]

nature portfolio

Corresponding author(s): Alexandra Kravchenko

Last updated by author(s): 04/06/2024

Reporting Summary

Nature Portfolio wishes to improve the reproducibility of the work that we publish. This form provides structure for consistency and transparency in reporting. For further information on Nature Portfolio policies, see our Editorial Policies and the Editorial Policy Checklist.

Statistics

For all statistical analyses, confirm that the following items are present in the figure legend, table legend, main text, or Methods section.

n/a	Confirmed	
☐	☒	The exact sample size (*n*) for each experimental group/condition, given as a discrete number and unit of measurement
☐	☒	A statement on whether measurements were taken from distinct samples or whether the same sample was measured repeatedly
☐	☒	The statistical test(s) used AND whether they are one- or two-sided<br>*Only common tests should be described solely by name; describe more complex techniques in the Methods section.*
☒	☐	A description of all covariates tested
☐	☒	A description of any assumptions or corrections, such as tests of normality and adjustment for multiple comparisons
☐	☒	A full description of the statistical parameters including central tendency (e.g. means) or other basic estimates (e.g. regression coefficient) AND variation (e.g. standard deviation) or associated estimates of uncertainty (e.g. confidence intervals)
☐	☒	For null hypothesis testing, the test statistic (e.g. *F*, *t*, *r*) with confidence intervals, effect sizes, degrees of freedom and *P* value noted<br>*Give P values as exact values whenever suitable.*
☒	☐	For Bayesian analysis, information on the choice of priors and Markov chain Monte Carlo settings
☐	☒	For hierarchical and complex designs, identification of the appropriate level for tests and full reporting of outcomes
☒	☐	Estimates of effect sizes (e.g. Cohen's *d*, Pearson's *r*), indicating how they were calculated

Our web collection on statistics for biologists contains articles on many of the points above.

Software and code

Policy information about availability of computer code

Data collection	No software was used for data collection. Data analysis: The raw amplicon sequences were analyzed by Mothur using Mothur Miseq SOP (accessed August 2021). The prediction of microbial functions was conducted by PICRUSt2. The downstream analyses were conducted in R (version 4.0.2) with RStudio (version 1.5042) using packages phyloseq (version 1.34.0), file2meco (version 0.1.0), microeco (version 0.5.1), microbiome (version 1.12.0), ampvis2 (version 2.7.11), vegan (version 2.5.7), pairwiseAdonis (version 0.4), circlize (version 0.4.13), dplyr (version 1.0.7), tidyr (version 1.1.4), ggpubr (version 0.4.0), rstatix (version 0.7.0) and ggplot2 (version 3.3.5).The Statistical analyses were conducted using proc mixed procedure of SAS software. All the detailed description of the above analyses are provided in the Methods.
Data analysis	Statistical analyses were conducted using proc mixed procedure of SAS software. Detailed description of the analyses are provided in the Methods.

For manuscripts utilizing custom algorithms or software that are central to the research but not yet described in published literature, software must be made available to editors and reviewers. We strongly encourage code deposition in a community repository (e.g. GitHub). See the Nature Portfolio guidelines for submitting code & software for further information.

Data

Policy information about availability of data

All manuscripts must include a data availability statement. This statement should provide the following information, where applicable:

- Accession codes, unique identifiers, or web links for publicly available datasets
- A description of any restrictions on data availability
- For clinical datasets or third party data, please ensure that the statement adheres to our policy

The sequencing data of the total DNA and SIP fractions were submitted to NCBI under Bioproject PRJNA801760 (accession numbers SAMN25378717 to SAMN25378796) and Bioproject PRJNA802612 (accession numbers SAMN25563888 to SAMN25564655), respectively. The sequences were aligned using SILVA bacteria database (Release 138) obtained from the Miseq SOP (accessed August 2021). All the data is publicly released.

Research involving human participants, their data, or biological material

Policy information about studies with human participants or human data. See also policy information about sex, gender (identity/presentation), and sexual orientation and race, ethnicity and racism.

Reporting on sex and gender	The study does not involve hum an participants.
Reporting on race, ethnicity, or other socially relevant groupings	The study does not involve hum an participants.
Population characteristics	The study does not involve hum an participants.
Recruitment	The study does not involve hum an participants.
Ethics oversight	The study does not involve hum an participants.

Note that full information on the approval of the study protocol must also be provided in the manuscript.

Field-specific reporting

Please select the one below that is the best fit for your research. If you are not sure, read the appropriate sections before making your selection.

☐ Life sciences ☐ Behavioural & social sciences ☒ Ecological, evolutionary & environmental sciences

For a reference copy of the document with all sections, see nature.com/documents/nr-reporting-summary-flat.pdf

Ecological, evolutionary & environmental sciences study design

All studies must disclose on these points even when the disclosure is negative.

Study description	The study analyzed soil and soil microbial characteristics from a replicated field experiment. The experiment has a factorial treatment deign with plant systems, incubation times, and pores sizes as the studied factors and their interactions. The experimental units are experimental plots and intact soil cores. Detailed description of the experimental design and replications are provided in the manuscript.
Research sample	A set of intact soil cores and adjacent bulk soil samples collected from individual plots of the field experimental site. Soils from bare system, monoculture switchgrass and high plant diversity prairie system were chosen to represent a range of plant diversities.
Sampling strategy	The sampling locations within individual experimental plots were selected at random. Three experimental plots per switchgrass and prairie system and four plots per bare system was sampled. In each plot six intact soil cores (5 cm Ø, 2.5 cm height) were collected from the 5 to 7.5 cm depth increment using a 5.7 cm diameter soil core sampler. The intact cores collected at random from each plot were in close proximity (<3 cm distance) to each other. These sample sizes and sampling approaches are consistent with the general practice of agricultural field experiments.
Data collection	The data were collected following the standardized established protocols and procedures for soil and microbial measurements. Soil data were collected by Kravchenko and Guber. Zheng Li collected the biological data. During the SIP process, refractive index values were measured using AR200 digital refractometer. Sampling was conducted in fall of 2020.
Timing and spatial scale	A single sampling event took place in the fall of 2020.
Data exclusions	No data were excluded from the analysis.
Reproducibility	All attempts to repeat the experiment were successful. We showed reproducibility in multiple phase of the experiment. For each

Reproducibility	total DNA and ultracentrifugation fraction, triplicates were submitted for sequencing.
Randomization	The treatments in the studied experimental field site were assigned to randomly selected replicated experimental plots in a randomized complete block design.
Blinding	To ensure blinding the soil samples were given ID numbers for processing.

Did the study involve field work? ☒ Yes ☐ No

Field work, collection and transport

Field conditions	Field sampling was conducted at temperature of 15C and no precipitation.
Location	Long Term Ecological Research (LTER) site, KBS, Hickory Corners, Michigan, USA (42o 24'N, 85o 23'W; 288-m elevation)
Access & import/export	The sampling was done after obtaining the approval of the LTER KBS, upon submission of the KBS GLBRC Site Use Request Form, approved by KBS LTER Executive Committee on 11/05/2020.
Disturbance	The sampling caused no disturbance.

Reporting for specific materials, systems and methods

We require information from authors about some types of materials, experimental systems and methods used in many studies. Here, indicate whether each material, system or method listed is relevant to your study. If you are not sure if a list item applies to your research, read the appropriate section before selecting a response.

Materials & experimental systems

n/a	Involved in the study	
☒	☐	Antibodies
☒	☐	Eukaryotic cell lines
☒	☐	Palaeontology and archaeology
☒	☐	Animals and other organisms
☒	☐	Clinical data
☒	☐	Dual use research of concern
☐	☒	Plants

Methods

n/a	Involved in the study	
☒	☐	ChIP-seq
☒	☐	Flow cytometry
☒	☐	MRI-based neuroimaging

Dual use research of concern

Policy information about dual use research of concern

Hazards

Could the accidental, deliberate or reckless misuse of agents or technologies generated in the work, or the application of information presented in the manuscript, pose a threat to:

No	Yes	
☒	☐	Public health
☒	☐	National security
☒	☐	Crops and/or livestock
☒	☐	Ecosystems
☒	☐	Any other significant area

Experiments of concern

Does the work involve any of these experiments of concern:

No	Yes	
☒	☐	Demonstrate how to render a vaccine ineffective
☒	☐	Confer resistance to therapeutically useful antibiotics or antiviral agents
☒	☐	Enhance the virulence of a pathogen or render a nonpathogen virulent
☒	☐	Increase transmissibility of a pathogen
☒	☐	Alter the host range of a pathogen
☒	☐	Enable evasion of diagnostic/detection modalities
☒	☐	Enable the weaponization of a biological agent or toxin
☒	☐	Any other potentially harmful combination of experiments and agents

Plants

Seed stocks	The was no seed planting involved in this study.
Novel plant genotypes	No novel genotypes were generated in this study.
Authentication	No novel genotypes were generated in this study.
